# Sixteen Novel Mycoviruses Containing Positive Single-Stranded RNA, Double-Stranded RNA, and Negative Single-Stranded RNA Genomes Co-Infect a Single Strain of *Rhizoctonia zeae*

**DOI:** 10.3390/jof10010030

**Published:** 2023-12-31

**Authors:** Siwei Li, Zhihao Ma, Xinyi Zhang, Yibo Cai, Chenggui Han, Xuehong Wu

**Affiliations:** College of Plant Protection, China Agricultural University, Haidian District, Beijing 100193, China; lisiwei28@163.com (S.L.); mzh622@cau.edu.cn (Z.M.); zxy18511290531@163.com (X.Z.); caiyibo_zb203@163.com (Y.C.); hanchenggui@cau.edu.cn (C.H.)

**Keywords:** sixteen novel mycoviruses, co-infection, *Rhizoctonia zeae*, metatranscriptome, rapid amplification of cDNA ends

## Abstract

In the present study, sixteen novel RNA mycoviruses co-infecting a single strain of *Rhizoctonia zeae* (strain D40) were identified and molecularly characterized using metatranscriptome sequencing combined with a method for rapid amplification of cDNA ends. The fungal strain was isolated from diseased seedlings of sugar beet with damping-off symptoms. Based on genome analysis and phylogenetic analysis of amino acid sequences of RNA-dependent RNA polymerase, the sixteen mycoviruses associated with strain D40 contained three genome types with nine distinct lineages, including positive single-stranded RNA (*Hypoviridae*, *Yadokariviridae*, *Botourmiaviridae*, and *Gammaflexiviridae*), double-stranded RNA (Phlegiviridae, *Megabirnaviridae*, Megatotiviridae, and Yadonushiviridae), and negative single-stranded RNA (*Tulasviridae*), suggesting a complex composition of a mycoviral community in this single strain of *R. zeae* (strain D40). Full genome sequences of six novel mycoviruses and the nearly full-length sequences of the remaining ten novel mycoviruses were obtained. Furthermore, seven of these sixteen mycoviruses were confirmed to assemble virus particles present in the *R. zeae* strain D40. To the best of our knowledge, this is the first detailed study of mycoviruses infecting *R. zeae*.

## 1. Introduction

Mycoviruses are viruses infecting fungi and are widespread in major taxa of fungi [1]. After the discovery of the first mycovirus in the edible mushroom *Agaricus bisporus* in 1962, numerous thorough studies were performed to explore the diversity of mycoviruses in the kingdom of fungi, including phytopathogenic, medical, endophytic, entomopathogenic, edible, and biocontrol fungi [2,3,4,5,6]. According to the latest ICTV taxonomy report and accepted list of all mycoviruses in the ICTV Master Species List 2022 MSL38.v3 (https://ictv.global/msl, accessed on 11 September 2023), there are 30 families and two genera consisting of a total of 491 mycovirus species. The majority of mycoviruses reported have RNA genomes, including twelve families (*Alphaflexiviridae*, *Barnaviridae*, *Botourmiaviridae*, *Deltaflexiviridae*, *Endornaviridae*, *Fusariviridae*, *Gammflexiviridae*, *Hadakaviridae*, *Hypoviridae*, *Mitoviridae*, *Narnaviridae*, and *Yadokariviridae*) with positive single-stranded RNA (+ssRNA) genomes, ten families (*Alternaviridae*, *Amalgaviridae*, *Chrysoviridae*, *Curvulaviridae*, *Megabirnaviridae*, *Partitiviridae*, *Polymycoviridae*, *Quadriviridae*, *Spinareoviridae*, and *Totiviridae*) and one genus (*Botybirnavirus*) with double-stranded RNA (dsRNA) genomes, five families (*Discoviridae*, *Mymonaviridae*, *Phenuiviridae*, *Rhabdoviridae*, and *Tulasviridae*) with negative single-stranded RNA (−ssRNA) genomes, and two families (*Metaviridae* and *Pseudoviridae*) with reverse transcribing RNA (RT-RNA) genomes; one family with an ssDNA genome (*Genomoviridae*) and one genus with a dsDNA genome (*Rhizidioivirus*) have also been reported to accommodate mycoviruses (https://ictv.global/msl, accessed on 11 September 2023).

Co-infection or mixed infection (a single strain infected with two or more related or unrelated viruses) is a common feature of mycovirus infection [7]. Metatranscriptome sequencing is a possibly unbiased virus-hunting tool that can further explore +ssRNA, dsRNA, −ssRNA, and ssDNA viruses in fungi [8,9]. Using the metatranscriptome sequence method, the co-infection event was found to be common in diverse fungi [2,10,11,12,13,14,15]. For example, nine mycoviruses were identified through metatranscriptome sequencing in a single strain SX276 of *Sclerotinia sclerotiorum* and assigned to eight potential families [12]; sixteen mycoviruses were found by metatranscriptome sequencing from a single strain MAFF 240,374 of *Fusarium poae*, harboring dsRNA, +ssRNA, and −ssRNA viruses assigned to more than eight families [13]; and seventeen viral species within eight families were discovered by metatranscriptome sequencing in an avirulent strain DC17 of *Rhizoctonia solani* [2].

*Rhizoctonia* is a soil-borne plant pathogen with a wide host range and causes various important crop diseases that result in significant economic losses, including diseases of rice sheath blight, maize banded leaf and sheath blight, potato black scurf and stem canker, and sugar beet seedling damping-off and root rot [16,17,18,19,20]. It is reported that *Rhizoctonia* hosts a large range of viruses, but some of them are still unclassified [16]. The first mycovirus in *Rhizoctonia* is reported by Butler and Castano [21]. Since then, numerous research studies related to mycoviruses associated with *Rhizoctonia* and the virus diversity in this pathogenic genus have been published [2,16]. To date, multiple mycoviruses have been reported to infect *Rhizoctonia*; most of them have accommodated the +ssRNA genome, followed by the dsRNA and −ssRNA genomes [16]. Among the mycoviruses associated with *Rhizoctonia*, some of them belong to well-studied families, including *Barnaviridae*, *Botourmiaviridae*, *Deltaflexiviridae*, *Endornaviridae*, *Hypoviridae*, *Megabirnaviridae*, *Mitoviridae*, *Narnaviridae*, *Fusariviridae*, and *Partitiviridae*, while some of them belong to proposed families or unclassified ones [16,22,23].

In this study, using the metatranscriptome sequencing method combined with rapid amplification of cDNA ends (RACE) and Sanger sequencing, sixteen novel mycoviruses (seven +ssRNA viruses, seven dsRNA viruses, and two −ssRNA viruses) were identified and characterized to co-infect *R. zeae* strain D40. Full-length genomes of six (RzHV1, RzOLV1, RzOLV2, RzRV1, RzRV2, and RzBYV1) of the 16 novel mycoviruses were obtained. Moreover, the genomic and phylogenetic properties of the 16 new mycoviruses present in *R. zeae* strain D40 were studied.

## 2. Materials and Methods

### 2.1. Extraction and Purification of RNA

Strain D40 was isolated from diseased seedlings of sugar beet with the symptom of damping-off and identified to be *R. zeae* using previously reported method [19,20,24]. Samples were collected from Qiqihaer city, Heilongjiang province of China, in 2010. Mycelia of D40 strain was cultured on potato dextrose agar (PDA) plates with cellophane film membranes (PDA-CF) at 25 °C in the dark for five days before extracting total RNA and dsRNA. Total RNA was extracted using RNAiso Plus (TaKaRa, Dalian, China) according to the manufacturer’s instructions. DsRNA of strain D40 was extracted using the CF-11 cellulose (Sigma-Aldrich, St. Louis, MI, USA) chromatography method, as previously described [25]. In order to remove polysaccharides and pigment from mycelia, addition of Fruit-mate™ for RNA Purification (TaKaRa) and High-Salt Solution for Precipitation (Plant) (TaKaRa) was conducted during extraction of both total RNA and dsRNA according to the manufacturer’s instructions.

### 2.2. Metatranscriptome Sequencing

Metatranscriptome sequencing of *R. zeae* strain D40 was carried out by Shanghai Biotechnology Corporation using an Illumina NovaSeq 6000 platform for paired-end sequencing. Sequencing libraries were established from rRNA-depleted total RNA samples of strain D40 using a Zymo-Seq RiboFree Total RNA Library Prep Kit (Zymo Research, California, CA, USA). Library quality assessment was determined on a Qubit^®^ 2.0 Fluorometer (Invitrogen, Carlsbad, CA, USA) and Agilent Technologies 2100 Bioanalyzer (Agilent Technologies, Santa Clara, CA, USA). Low-quality reads and fungi mRNA sequences were filtered out from the resulting raw reads, and thus high-quality clean reads were obtained. A de novo assembly of the primary unigenes was constructed using CLC Genomics Workbench version 6.0.4 software. Final unigenes were assembled using CAP3 EST software. The resulting final unigenes were queried against the National Center for the Biotechnology Information (NCBI) non-redundant (NR) database and aligned using BLASTx to obtain homologous sequences of mycoviruses. The unmatched contigs, both with size of nucleotides over 1.5 kb and number of reads over 1000, were kept [26].

### 2.3. Validation of Mycoviruses in Strain D40

To verify the presence of putative mycoviruses in strain D40, reverse transcription polymerase chain reaction (RT-PCR) was performed using specific primers (listed in Table S1) designed according to the results of metatranscriptome sequencing. To confirm the viral sequences, especially for the less assembled region with obscure N bases and/or fragments/gaps from certain viral contigs, amplification using specific primers (listed in Table S2) designed based on the de novo assembled sequences was conducted. Total RNA of strain D40 was reversely amplified to synthesize first-strand complementary DNA (cDNA) using Moloney murine leukemia virus (M-MLV) reverse-transcriptase and random primer pd(N)_6_ (TaKaRa) according to the manufacturer’s instructions. The amplified products were used as a template in subsequent RT-PCR amplifications. The PCR was performed in a total volume of 25 μL, containing 12.5 μL 2× Power *Taq* PCR MasterMix (0.1 U/μL DNA polymerase, 3 mM Mg^2+^ plus buffer, and 500 μM dNTP) (Beijing Tianyihuiyuan Co., Ltd., Beijing, China), 9.5 μL deionized water, 1 μL of primer pair (10 μM), and 1 μL cDNA template. RT-PCR was performed in an Eppendorf Mastercycler gradient thermal cycler (Eppendorf, Hamburg, Germany) using the following program: initial denaturation at 95 °C for 3 min, followed by 35 cycles of denaturing at 95 °C for 30 s, annealing at temperature of the primer pairs (listed in Tables S1 and S2) for 30 s, extension at 72 °C for 1 min, and a final extension at 72 °C for 10 min. After examination of amplified product with 1% agarose gel electrophoresis, target bands were cut and purified using a gel extraction kit (Aidlab Biotechnologies, Beijing, China). All the amplified products mentioned above were cloned into pClone007 Versatile Simple Vector (TsingKe, Beijing, China) and transformed into *Escherichia coli* Trelief™ 5α cells (TsingKe) and sequenced by Sanger sequencing (TsingKe). The amplicons were sequenced and compared with the metatranscriptome-derived sequences.

### 2.4. Determination of Full-Length cDNAs of Putative Mycoviruses

The 5′- and 3’-terminal sequences of the putative mycoviruses were determined by RACE method using a SMARTer RACE 5′/3′ Kit (TaKaRa). The 5′-RACE was performed using the SMARTer RACE 5′/3′ Kit as instructed. The 3′-RACE was performed through addition of a polyA tail to the RNA fragments using the polyA polymerase (TaKaRa) followed by an oligo-dT-adapter primer. A series of nested PCR amplifications were then performed using gene-specific primers (GSPs) and Universal Primer Mix (UPM). GSPs used to amplify 5′- and 3′-terminal sequences are listed in Table S3. The amplified products were cloned into pClone007 Versatile Simple Vector (TsingKe) and transformed into *E. coli* Trelief™ 5α cells (TsingKe) and sequenced by Sanger sequencing (TsingKe). The identity of each base was determined using at least three independent clones that were sequenced in both directions.

### 2.5. Sequence Analysis and Phylogenetic Analysis

The complete cDNA sequence of each mycovirus was assembled by combining the metatranscriptome sequences with gap sequences and/or terminal sequences using DNAMAN software 7.0 (Lynnon Biosoft, CA, USA). The confirmed complete genome sequences of six mycoviruses and partial genome sequences of ten mycoviruses were submitted to NCBI database. Viral sequences of each virus assembled from the metatranscriptome data, gap sequences, and terminal sequences were used as queries to search the NCBI non-redundant protein database of GenBank using BLASTx. ORF predictions were performed using ORF finder (https://www.ncbi.nlm.nih.gov/orffinder/, accessed on 11 September 2023) from NCBI, and predictions were made by selecting the “standard” genetic code for all viral contigs. The molecular mass of protein encoded by each ORF was predicted by DNAMAN software 7.0. The predicted amino acid (aa) sequences were analyzed through a BLASTp search with standard settings. The putative function of ORF was predicted based on the result of BLASTp best match. Conserved domains were identified using the conserved domain database (https://www.ncbi.nlm.nih.gov/cdd/, accessed on 11 September 2023).

CLUSTAL_X was used to perform multiple alignments of aa sequences of RdRp or helicase domains [27]. The maximum likelihood (ML) phylogenetic trees were constructed using the IQ-TREE version 2.2.2.6 software with 1000 ultrafast bootstrap replicates, and the best-fit amino acid substitution models were identified using ModelFinder [28,29,30]. Phylogenetic trees were visualized in FigTree version 1.4.4 software (http://tree.bio.ed.ac.uk/software/figtree/, accessed on 11 September 2023).

### 2.6. Extraction of Virions and Confirmation of Mycoviruses That Assemble Virions

*R. zeae* strain D40 was cultured on PDA-CF plates for five days in the dark at 25 °C. Approximately 10 g of fresh mycelia were collected and ground to a fine powder in liquid nitrogen. The mycelial powder was then used to extract virus particles as previously described [23]. The obtained virus particles were suspended in 100 µL phosphate-buffered saline (PBS, 0.01 M, pH 7.2), stained with 2% (*w*/*v*) uranyl acetate, and observed with a transmission electron microscope (TEM) (HT7800, HITACHI, Tokyo, Japan).

To confirm which virus was responsible for assembling virions, total RNA was extracted from the virion suspension using TRIzol Reagent (Aidlab Biotechnologies) to synthesize first-strand cDNA according to the methods described in Section 2.3. The resulting PCR product was then used as a template for RT-PCR. RT-PCR was conducted using specific primers (listed in Table S1) designed based on the viral contigs.

## 3. Results

### 3.1. Identification of Mycoviruses Present in R. zeae Strain D40

**Figure 1:**
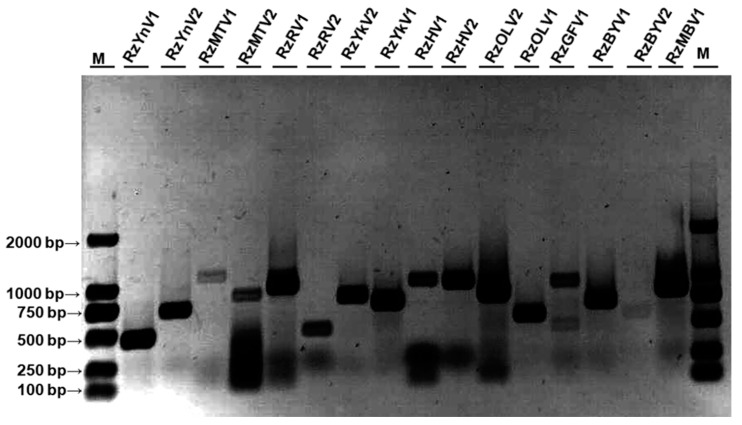
Identification of mycoviruses associated with *Rhizoctonia zeae* strain D40. Confirmation of mycoviruses present in strain D40 by reverse transcription polymerase chain reaction (RT-PCR). Products amplified by RT-PCR were visualized by 1% agarose gel electrophoresis. M: DNA marker DL2000.

Multiple bands of dsRNA extracted from *R. zeae* strain D40 were visualized via electrophoresis, indicating the presence of multiple dsRNA mycoviruses and/or replicative intermediates of ssRNA mycoviruses (Figure S1). Metatranscriptome sequencing on an Illumina NovaSeq 6000 platform was conducted to characterize the mycovirome associated with *R. zeae* strain D40, resulting in a total of 53,737,284 raw reads with 8.06 Gb sequence information. After filtering out 3.7% of raw reads, the resulting clean reads were de novo assembled into 30,032 contigs with length >200 nt. Raw data were deposited into the Sequence Read Archive (SRA) under the accession number SAMN33386006. After filtering, contigs, both with a size of nucleotides over 1.5 kb and a number of reads over 1000, were kept, and sixteen viral sequences were further assembled and confirmed to be present in *R. zeae* strain D40 by RT-PCR (Figure 1).

Complete or partial genomic sequences were obtained by metatranscriptome sequencing, completed by Sanger sequencing of gaps, when possible, as well as by determination of RNA ends. Based on the results of virus validation, sixteen mycoviruses putatively identified to be present in strain D40 belonged to nine taxonomic lineages, namely *Hypoviridae*, *Yadokariviridae*, *Botourmiaviridae*, *Gammaflexiviridae*, Phlegiviridae, *Megabirnaviridae*, Megatotiviridae, Yadonushiviridae, and *Tulasviridae* (Table 1).

### 3.2. Genome Organization and Phylogenetic Analysis of Putative Members of the Family Hypoviridae

**Figure 2:**
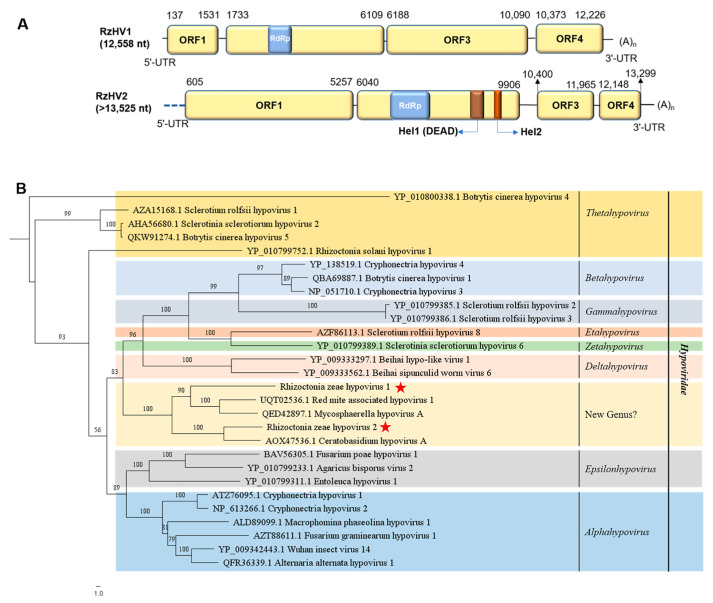
Genome organization of Rhizoctonia zeae hypovirus 1 (RzHV1) and Rhizoctonia zeae hypovirus 2 (RzHV2) and maximum likelihood tree depicting the relationships of the predicted amino acid (aa) sequences of RNA-dependent RNA polymerase (RdRp) of assembled sequences with identities to members of the family *Hypoviridae*. (**A**) Genome organizations of RzHV1 and RzHV2. Open reading frames (ORFs) are shown as boxes. Three conserved domains in RdRp, DEAD/DEAH box helicase (Hel1 (DEAD)), and SF2_C_RHA (Hel2) are shown in blue box, borrow box, and red box, respectively. (**B**) Maximum likelihood phylogenetic tree was constructed based on the aa sequences of RdRp of RzHV1, RzHV2, along with 26 representative hypoviruses using IQ-TREE with the best-fit model “VT+F+R5”. Numbers on the branches indicate the percentage of bootstrap support from 1000 replicates. The red stars indicate the position of RzHV1 and RzHV2.

Sequences of contig201 and contig1743 assembled from metatranscriptome data were identified to be two new members of the family *Hypoviridae*, which were named Rhizoctonia zeae hypovirus 1 (RzHV1) and Rhizoctonia zeae hypovirus 2 (RzHV2), respectively. The whole genome sequence of RzHV1, whose full genome was 12,588 nt in size with a 51.7% G+C content (GenBank accession no. OQ559666), excluding a polyA tail, was obtained using the data of metatranscriptome sequencing combined with the RACE method. The 5′- and 3′-UTRs of RzHV1 were 136 nt and 332 nt in length, respectively. RzHV1 contains four predicted ORFs (Figure 2A): ORF1 (137–1531 nt), ORF2 (1733–6109 nt), ORF3 (6188–10,090 nt), and ORF4 (10,373–12,226 nt). ORF1 encodes a putative protein of 464 aa (molecular mass: 52.708 kDa) in length, ORF2 encodes RdRp of 1458 aa (molecular mass: 162.997 kDa) in length with a conserved domain of ps-ssRNAv_Hypoviridae_RdRp (cd23170, 2.23 × 10^−17^), and ORF3 and ORF4 encode putative proteins of 1300 aa (molecular mass: 146.728 kDa) and 617 aa (molecular mass: 68.582 kDa) in length, respectively.

RzHV2 was identified from metatranscriptome sequencing with a near full-length genome of 13,525 nt (GenBank accession no. OQ559672) excluding the polyA tail, which contains four predicted ORFs (Figure 2A): ORF1 (605–5257 nt), ORF2 (6040–9906 nt), ORF3 (10,400–11,965 nt), and ORF4 (12,148–13,299 nt). ORF1 encodes a putative protein of 1550 aa (molecular mass: 171.462 kDa) in length; ORF2 encodes a polyprotein of 1288 aa (molecular mass: 167.771 kDa) in length with three conserved domains, namely ps-ssRNAv_Hypoviridae_RdRp (cd23170, 1.46 × 10^−17^), DEAD/DEAH box helicase (Hel1) (pfam00270, 1.89 × 10^−03^), and SF2_C_RHA: C-terminal helicase domain of the RNA helicase A (Hel2) (cd18791, 6.05 × 10^−10^); and ORF3 and ORF4 encode putative proteins of 521 aa (molecular mass: 58.649 kDa) and 383 aa (molecular mass: 43.028 kDa) in length, respectively.

A homology search with BLASTp showed that the complete aa sequence of RdRp encoded by ORF2 of RzHV1 and RzHV2 were the most closely related to that of RdRp of Mycosphaerella hypovirus A (31.50% identity) and Ceratobasidium hypovirus A (46.14% identity), respectively. However, ORF1 and ORF4 of RzHV1 or ORF3 and ORF4 of RzHV2 showed no significant sequence identity with proteins in the database of NCBI using a BLASTp search. The RdRp domain of RzHV1 and RzHV2 contains eight conserved motifs (I–VIII) with a GDD tripeptide within motif VI (Figure S2A). The Hel domains (Hel1 and Hel2) of RzHV2 contain six conserved motifs (I–VI) with a DEAH tetrapeptide within motif III (Figure S2B).

To understand the phylogenetic relationships among RzHV1, RzHV2, and 26 other related viruses, phylogenetic analysis based on the aa sequences of RdRp of hypoviruses was performed (Figure 2B). These 28 hypoviruses were classified into nine groups, including eight genera (*Alphahypovirus*, *Betahyovirus*, *Gammahypovirus*, *Deltahypovirus*, *Epsilonhypovirus*, *Zetahypovirus*, *Thetahypovirus*, and *Etahypovirus*) of the family *Hypoviridae* (Figure 2B). RzHV1 and RzHV2 are distant from these eight genera mentioned above and form an independent phylogenetic group. RzHV1 and RzHV2 clustered with red-mite-associated hypovirus 1, Mycosphaerella hypovirus A, and Ceratobasidium hypovirus A. Taken together, on the basis of the unique genome organization of four ORFs, low identities of BLASTp, and phylogenetic position, one new genus was proposed to be established in the family *Hypoviridae* to accommodate RzHV1, RzHV2, and other related hypoviruses.

### 3.3. Genome Organization and Phylogenetic Analysis of Putative Members of the Family Yadokariviridae

**Figure 3:**
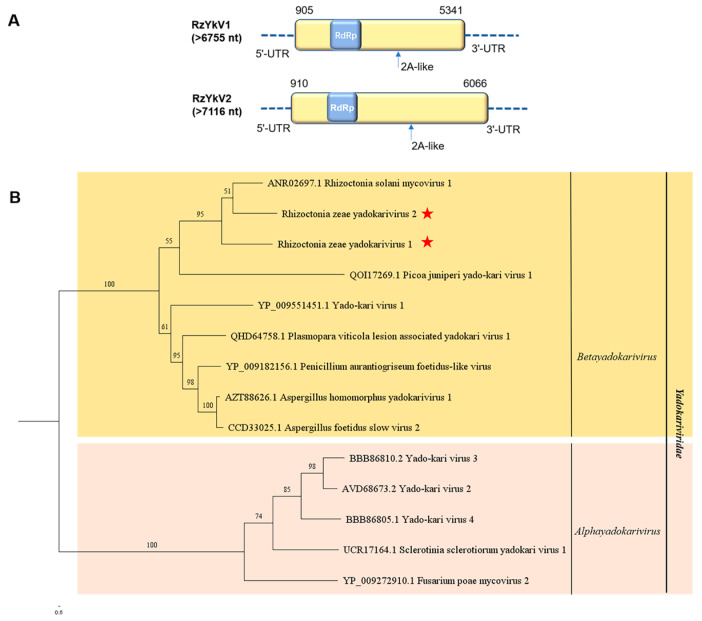
Genome organization of Rhizoctonia zeae yadokarivirus 1 (RzYkV1) and Rhizoctonia zeae yadokarivirus 2 (RzYkV2) and maximum likelihood tree depicting the relationships of the predicted amino acid (aa) sequences of RNA-dependent RNA polymerase (RdRp) of assembled sequences with identities to members of the family *Yadokariviridae*. (**A**) Genome organizations of RzYkV1 and RzYkV2. Open reading frames (ORFs) are shown as boxes. One conserved domain in RdRp is shown in blue box. (**B**) Maximum likelihood phylogenetic tree was constructed based on the aa sequences of RdRp of RzYkV1, RzYkV2, and 12 other representative yadokariviruses using IQ-TREE with the best-fit model “VT+F+R4”. Numbers on the branches indicate the percent bootstrap support from 1000 replicates. The red stars indicate the position of RzYKV1 and RzYKV2.

Contig764 and contig351 were identified from metatranscriptome sequencing with homologies of yadokariviruses, which were designated as Rhizoctonia zeae yadokarivirus 1 (RzYkV1) and Rhizoctonia zeae yadokarivirus 2 (RzYkV2), respectively. The near full-length genome sequences of RzYkV1 and RzYkV2 are 6775 nt (GenBank accession no. OQ559673) and 7116 nt (GenBank accession no. OQ559674) in length, respectively. Genomes of both RzYkV1 and RzYkV2 are composed of a single ORF, which encodes a protein of 1478 aa (molecular mass: 167.652 kDa) and a protein of 1718 aa (molecular mass: 194.131 kDa), with a conserved domain of RNA_dep_RNAP (cd01699, 9.72 × 10^−17^ and 1.05 × 10^−15^, respectively), respectively (Figure 3A). The results of a BLASTp search indicated that the complete aa sequences of RdRp of RzYkV1 and RzYkV2 shared the highest identities with that of Aspergillus homomorphus yadokarivirus 1 of 45.16% and 47.04%, respectively. Seven conserved motifs (A–G) with a GDD tripeptide within motif C were found in RdRp of RzYkV1 and RzYkV2 (Figure S3A). In addition, 2A-like motifs were discovered in RzYkV1 (GDVHPNPGP, 888–896 aa) and RzYkV2 (GDVHPNPGP, 1043–1051 aa), which are conserved in yadokariviruses (Figure S3B) [31].

To understand phylogenetic relationships among RzYkV1, RzYkV2, and 12 other related viruses, phylogenetic analysis based on aa sequences of RdRp of yadokariviruses was performed (Figure 3B). Selected yadokariviruses within the family *Yadokariviridae* were divided into two distinct clades, *Alphayadokarivirus* and *Betayadoakarivirus*. RzYkV1 and RzYkV2 were closely clustered with Rhizoctonia solani mycovirus 1 in the subclade of betayadokariviruses.

### 3.4. Genome Organization and Phylogenetic Analysis of Putative Members of the Family Botourmiaviridae

**Figure 4:**
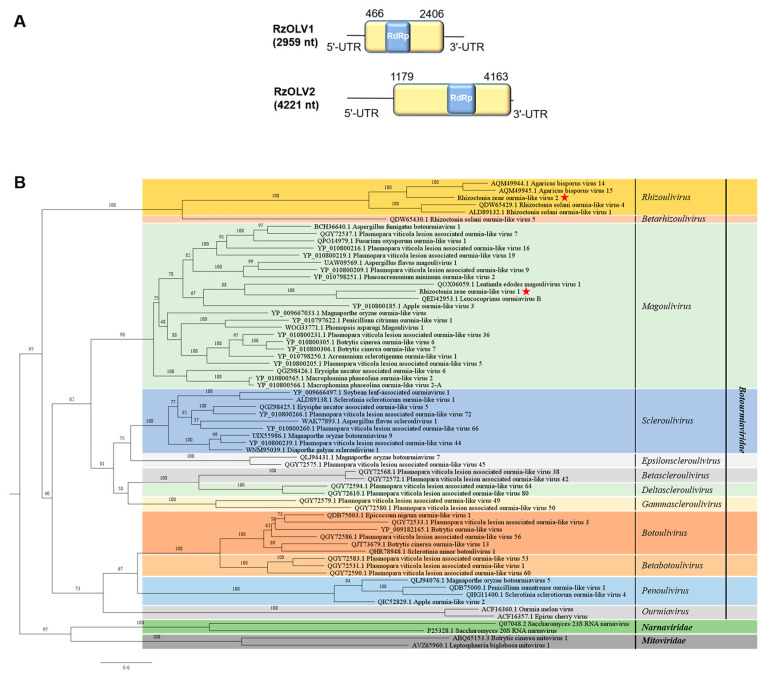
Genome organization of Rhizoctonia zeae ourmia-like virus 1 (RzOLV1) and Rhizoctonia zeae ourmia-like virus 2 (RzOLV2) and maximum likelihood tree depicting the relationships of the predicted amino acid (aa) sequences of RNA-dependent RNA polymerase (RdRp) of assembled sequences with identities to members of the three families, *Botourmiaviridae*, *Mitoviridae*, and *Narnaviridae*. (**A**) Genome organizations of RzOLV1 and RzOLV2. Open reading frames (ORFs) are shown as boxes. One conserved domain in ps-ssRNAv_Botourmiaviridae_RdRp is shown in blue box. (**B**) Maximum likelihood phylogenetic tree was constructed based on the aa sequences of RdRp of RzOLV1, RzOLV2, and 63 other representative viruses of the three families, *Botourmiaviridae*, *Mitoviridae*, and *Narnaviridae*, using IQ-TREE with the best-fit model “VT+F+R6”. Numbers on the branches indicate the percent bootstrap support from 1000 replicates. The red stars indicate the position of RzOLV1 and RzOLV2.

Two contigs (contig69 and contig132) obtained from metatranscriptome sequencing were identified to show homologies with botourmiaviruses using BLASTx, representing two new members of the family *Botourmiarviridae* and were named Rhizoctonia zeae ourmia-like virus 1 (RzOLV1) and Rhizoctonia zeae ourmia-like virus 2 (RzOLV2), respectively. The full-length genomes of RzOLV1 and RzOLV2 were amplified through serial RT-PCR amplification with specific primers (Table S2) to confirm the accuracy of the assembled sequences. RzOLV1 was identified with a full genome size of 2959 nt with a G+C content of 55.1% (GenBank accession no. OQ559667). The 5′- and 3′-UTRs of RzOLV1 were 465 nt and 553 nt in length, respectively. ORF prediction reveals that RzOLV1 consists of a single ORF of 1941 nt (466–2406 nt), which encodes a protein of 646 aa (molecular mass: 111.151 kDa) in length with a conserved domain of ps-ssRNAv_Botourmiaviridae_RdRp (cd23183, 2.15 × 10^−46^) (Figure 4A). The termini of RzOLV1 harbored a G-pentamer (GGGGG) at the 5′-terminus and a C-pentamer (CCCCC) at the 3′-terminus, which are also found in another ourmiavirus [32]. The full-length genome of RzOLV2 was 4221 nt with a 55.7% G+C content (GenBank accession no. OQ559668), which contained a single ORF of 2985 nt (1179–4163 nt) encoding a protein of 994 aa (molecular mass: 72.463 kDa) in length with a conserved domain of ps-ssRNAv_Botourmiaviridae_RdRp (cd23183, 1.39 × 10^−19^) (Figure 4A). The 5′- and 3′-UTRs of RzOLV2 were 1178 nt and 58 nt in length, respectively.

A homology search with BLASTp showed that the complete aa sequence of single ORF of RzOLV1 was the most closely (44.95% identity) related to that of RdRp of Leucocoprinus ourmiavirus B, while the complete aa sequence of single ORF of RzOLV2 was the most closely (43.65% identity) related to that of RdRp of Agaricus bisporus virus 15. Multiple alignments of the aa sequences of RzOLV1, RzOLV2, and seven other representative members in the family *Botourmiaviridae* showed that eight motifs (I–VIII) with a GDD tripeptide within motif VI were identified from RzOLV1 and RzOLV2 (Figure S4). A phylogenetic tree constructed based on aa sequences of RdRp of members within the three families, *Botourmiaviridae*, *Mitoviridae*, and *Narnaviridae*, showed that botourmiaviruses were divided into 12 major clades belonging to 12 classified genera (Figure 4B). RzOLV2 clustered with members of the genus *Rhizoulivirus*, while RzOLV1 clustered with members of the genus *Magoulivirus*.

### 3.5. Genome Organization and Phylogenetic Analysis of Putative Members of the Family Gammaflexiviridae

**Figure 5:**
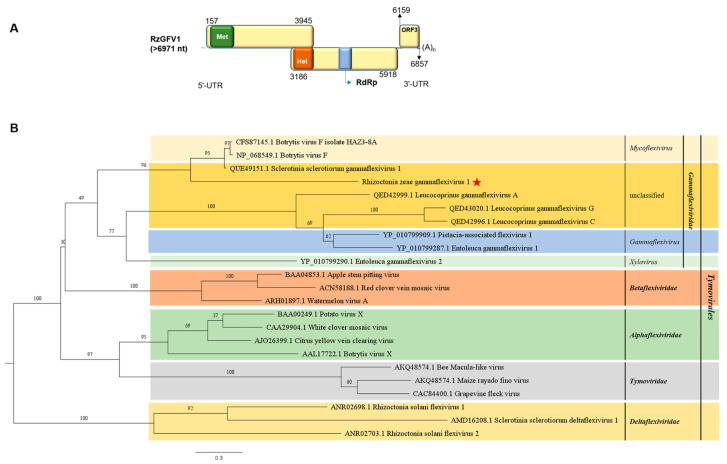
Genome organization of Rhizoctonia zeae gammaflexivirus 1 (RzGFV1) and maximum likelihood tree depicting the relationships of the predicted amino acid (aa) sequences of RNA-dependent RNA polymerase (RdRp) of assembled sequences with identities to members of the order *Tymovirales*. (**A**) Genome organization of RzGFV1. Open reading frames (ORFs) are shown as boxes. Three conserved domains in viral methltransferase (Met), viral_helicase1 (Hel), and RdRp_2 (RdRp) are shown in green box, orange box, and blue box, respectively. (**B**) Maximum likelihood phylogenetic tree was constructed based on the aa sequences of the core conserved domain of RdRp of RzGFV1 and 23 other representative viruses of the order *Tymovirales* using IQ-TREE with the best-fit model “Q.pfam+I+G4”. Numbers on the branches indicate the percent bootstrap support from 1000 replicates. The red star indicates the position of RzGFV1.

Contig797, assembled from metatranscriptome data, was identified to be a new member of the family *Gammaflexiviridae*, which was named Rhizoctonia zeae gammaflexivirus 1 (RzGFV1). The near full-length genome of RzGFV1 is 6971 nt (GenBank accession no. OQ559675), excluding the polyA tail. Three ORFs, ORF1 (157–3945 nt), ORF2 (3186–5918 nt), and ORF3 (6159–6857 nt), were predicted from RzGFV1 (Figure 5A), with ORF1 being overlapped with ORF2. ORF1 encodes a protein of 1262 aa (molecular mass: 135.275 kDa) in length, which contains one conserved domain of Vmethyltransf: viral methltransferase (Met) (pfam01660, 4.34 × 10^−38^); ORF2 encodes a polyprotein of 910 aa (molecular mass: 101.623 kDa) in length, which contains two conserved domains, including viral_helicase1 (Hel) (pfam01443, 1.64 × 10^−25^) and RdRp_2 (pfam00978, 5.66 × 10^−09^); and ORF3 encodes a protein of 232 aa in length (molecular mass: 25.666 kDa) with an unknown function.

Results of a BLASTp search showed that the complete aa sequence of ORF1 of RzGFV1 shared the highest identities (28.95%) with that of the replicase of Sclerotinia sclerotiorum gammaflexivirus 1, and the complete aa sequence of ORF2 of RzGFV1 was closest (34.77% identity) with that of the replicase of Botrytis virus F. However, ORF3 of RzGFV1 had no significant sequence identity with proteins in the database of NCBI using a BLASTp search. Multiple alignments of the aa sequences of RdRp of RzGFV1 and other gammaflexiviruses showed that eight motifs (I–VIII) with a GDD tripeptide within motif VI were identified from RzGFV1 (Figure S5). A phylogenetic tree constructed based on the aa sequences of RdRp of RzGFV1 and 23 other viruses within the order *Tymovirales* indicated that RzGFV1 clustered with members of the family *Gammaflexiviridae* in a separate subclade (Figure 5B).

### 3.6. Genome Organization and Phylogenetic Analysis of Putative Members of the Order Ghabrivirales

Nine contigs (contig75, contig620, contig7030, contig9453, contig4186, contig6273, contig496, contig3587, and contig3588) related to members of the order *Ghabrivirales* were identified from the metatranscriptome data.

**Figure 6:**
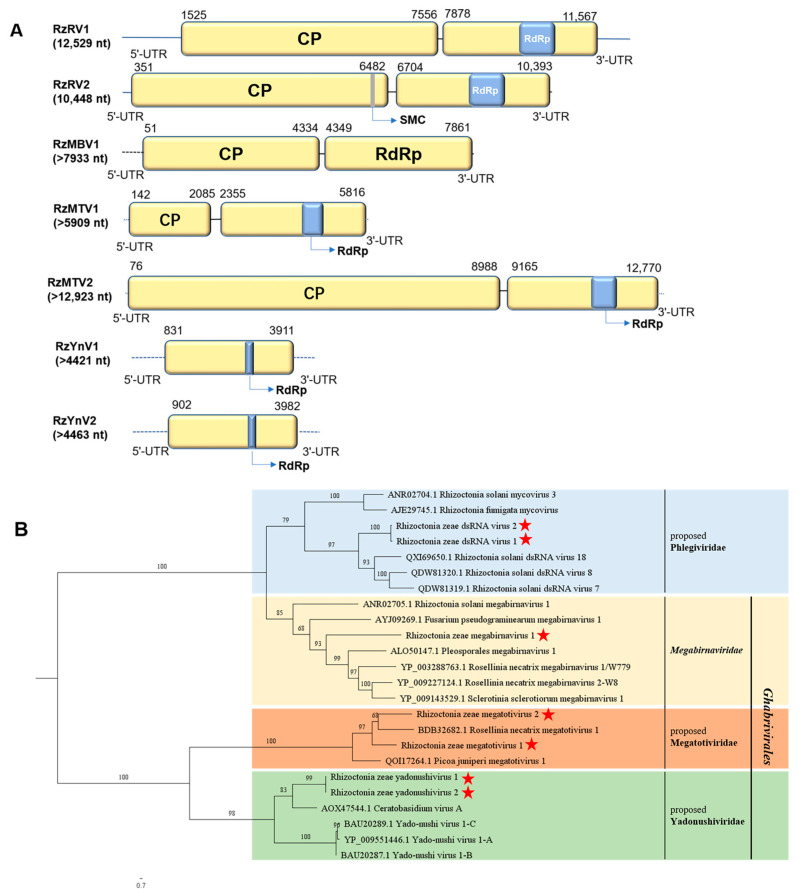
Genome organization of Rhizoctonia zeae megabirnavirus 1 (RzMBV1), Rhizoctonia zeae RNA virus 1 (RzRV1), Rhizoctonia zeae RNA virus 2 (RzRV2), Rhizoctonia zeae megatotivirus 1 (RzMTV1), Rhizoctonia zeae megatotivirus 2 (RzMTV2), Rhizoctonia zeae yadonushivirus 1 (RzYnV1), and Rhizoctonia zeae yadonushivirus 2 (RzYnV2) and maximum likelihood tree depicting the relationships of the predicted amino acid (aa) sequences of RNA-dependent RNA polymerase (RdRp) of assembled sequences with identities of members of the family *Megabirnaviridae* and three proposed families: Phlegiviridae, Megatotiviridae, and Yadonushiviridae. (**A**) Genome organizations of RzMBV1, RzRV1, RzRV2, RzMTV1, RzMTV2, RzMTV1, and RzMTV2. Open reading frames (ORFs) are shown as boxes. Two conserved domains in RdRp and SMC_pork_B (SMC) care are shown in blue box and gray box, respectively. (**B**) Maximum likelihood phylogenetic tree was constructed based on the aa sequences of RdRp of RzMBV1, RzRV1, RzRV2, RzMTV1, RzMTV2, RzYnV1, and RzYnV2 and six megabirnaviruses, five phlegiviruses, two megatotiviruses, and four yadonushiviruses using IQ-TREE with the best-fit model “Q.pfam+F+R4”. Numbers on the branches indicate the percent of bootstrap support from 1000 replicates. The red stars indicate the position of RzMBV1, RzRV1, RzRV2, RzMTV1, RzMTV2, RzYnV1, and RzYnV2.

Contig75 and contig620 were identified to be phylogenetically related to the members of the proposed family Phlegiviridae, which were named Rhizoctonia zeae RNA virus 1 (RzRV1) and Rhizoctonia zeae RNA virus 2 (RzRV2), respectively. The full length of RzRV1 is 12,529 nt with a G+C content of 53.6% (GenBank accession no. OQ559669). The 5′- and 3′-UTRs of RzRV1 were 1524 nt and 962 nt in length, respectively. Two ORFs, ORF1 (1525–7656 nt) and ORF2 (7878–11,567 nt), were predicted from RzRV1 (Figure 6A). ORF1 encodes a protein of 2043 aa (molecular mass: 222.141 kDa) in length; ORF2 encodes a protein of 1229 aa (molecular mass: 135.887 kDa) in length, which contains a conserved domain of RdRp_4 (pfam02123, 1.55 × 10^−19^). The full length of RzRV2 is 10,448 nt with 54.4% G+C content (GenBank accession no. OQ559670). The 5′- and 3′-UTRs of RzRV2 are 350 nt and 55 nt in length, respectively. Two ORFs, ORF1 (351–6482 nt) and ORF2 (6704–10,393 nt), were predicted from RzRV2. ORF1 encodes a protein of 2043 aa (molecular mass: 222.213 kDa) in length, which contains a conserved domain of SMC_pork_B (TIGR02168, 7.41 × 10^−04^); ORF2 encodes a protein of 1229 aa (molecular mass: 135.981 kDa) in length. Moreover, the ORF2 of these two viruses both contain a conserved domain of RdRp_4 (pfam02123, 1.34 × 10^−18^). Based on a BLASTp search, the complete aa sequences of ORF2 of both RzRV1 and RzRV2 shared the highest identities with that of RdRp of Rhizoctonia solani dsRNA virus 18 at 42.52% and 42.43%, respectively.

Contig7030, contig9453, and contig4186 were subjected to a BLASTx search, and the results indicated that these three contigs all exhibited homologies with megabirnaviruses. The gaps between contig7030, contig9453, and contig4186 were filled by RT-PCR with specific primers (listed in Table S2) designed based on the three contigs, which were assembled as a single viral sequence with the size of 7933 nt. We nominated this viral sequence of Rhizoctonia zeae megabirnavirus 1 (RzMBV1) and its near full-length genome and deposited them into NCBI (GenBank accession no. OQ559676). RzMBV1 contains two predicted ORFs (Figure 6A), ORF1 (51–4334 nt) and ORF2 (4349–7861 nt), with an intergenic region of 15 nt between the two ORFs. ORF1 is predicted to be 1427 aa (molecular mass: 153.632 kDa) long and has the highest identity of 28.02% to the hypothetical protein of Rhizoctonia solani megabirnavirus 2 using a BLASTp search; ORF2 encodes a protein of 1170 aa (molecular mass: 130.127 kDa) in length, which is the most closely (37.97% identity) related to RdRp of Pleosporales megabirnavirus 1.

Two contigs (contig6273 and contig496) were identified to share homologies with the members of the proposed family Megatotiviridae, which were designated as Rhizoctonia zeae megatotivirus 1 (RzMTV1) and Rhizoctonia zeae megatotivirus 2 (RzMTV2), respectively. The near full-length genome of RzMTV1 is 5909 nt (GenBank accession no. OQ559677), with two ORFs (ORF1 and ORF2) separated by an intergenic region (2086–2354 nt) (Figure 6A). ORF1 (142–2085 nt) encodes a protein of 647 aa (molecular mass: 72.243 kDa) in length; ORF2 (2355–5816 nt) encodes a protein of 1153 aa (molecular mass: 132.406 kDa) in length, which contains a conserved domain of RdRp_4 (pfam02123, 1.40 × 10^−05^). The near full-length genome of RzMTV2 is 12,923 nt (GenBank accession no. OQ559678) in length with two ORFs (ORF1 and ORF2) separated by an intergenic region (8989–9164 nt) (Figure 6A). ORF1 (76–8988 nt) encodes a protein of 2970 aa (molecular mass: 328.789 kDa) in length; ORF2 (9165–12,770 nt) encodes a protein of 1201 aa (molecular mass: 138.633 kDa) in length, which contains a conserved domain of RdRp_4 (pfam02123, 3.72 × 10^−11^). A homology search with BLASTp showed that the complete aa sequences of ORF1 of RzMTV1 and RzMTV2 were the most closely related to that of coat protein (CP) of Rosellinia necatrix megatotivirus 1 (31.20% and 27.68% identities, respectively), and the complete aa sequences of ORF2 of RzMTV1 and RzMTV2 were the most closely related to that of RdRp of Rosellinia necatrix megatotivirus 1 (45.05% and 41.68% identities, respectively).

Two contigs (contig3587 and contig3588) with high pairwise identity (86.56%) with each other were identified from metatranscriptome data and had the highest homologies with yadonushiviruses, which were tentatively named Rhizoctonia zeae yadonushivirus 1 (RzYnV1) and Rhizoctonia zeae yadonushivirus 2 (RzYnV2), respectively. Attempts to amplify the full sequences of complete ORF encoding CP of these two viruses were unsuccessful. Both of the partial sequences of RzYnV1 and RzYnV2 were predicted to contain one complete ORF encoding RdRp (Figure 6A). The partial sequence of RzYnV1 is 4421 nt (GenBank accession no. OQ559679) in length. One ORF (831–3911 nt) of 1026 aa (molecular mass: 116.961 kDa) is predicted from a partial sequence of RzYnV1, which contains a conserved domain of RdRp_4 (pfam02123, 1.80 × 10^−06^). The partial sequence of RzYnV2 is 4463 nt (GenBank accession no. OQ559680) in length. One ORF (902–3982 nt) of 1026 aa (molecular mass: 116.961 kDa) is predicted from a partial sequence of RzYnV2, which also contains a conserved domain of RdRp_4 (pfam02123, 1.72 × 10^−06^). A homology search with BLASTp showed that the complete aa sequences of ORF of RzYnV1 and RzYnV2 were the most closely related to that of RdRp of Ceratobasidium virus A with 49.41% and 49.62% identities, respectively.

Multiple alignments of aa sequences of RdRp of the seven dsRNA viruses (RzRV1, RzRV2, RzMBV1, RzMTV1, RzMTV2, RzYnV1, and RzYnV2) within the order *Ghabrivirales* showed that there were eight conserved motifs (I–VIII), seven of which had a tripeptide GDD in the motif VI (Figure S6). To identify the phylogenetic status of the seven mycoviruses related to the order *Ghabrivirales*, a phylogenetic tree was constructed based on the complete aa sequences of RdRp of these seven mycoviruses and 17 other ghabriviruses (Figure 6B). As a result, RzRV1 and RzRV2 clustered with members in the proposed family Phlegiviridae in a separate subclade; RzMBV1 clustered with Pleosporales megabirnavirus 1, Rosellinia necatrix megabirnavirus 1/W779, Sclerotinia sclerotiorum megabirnavirus 1, and Rosellinia necatrix megabirnavirus 2-W8 in a separate subclade; RzMTV1 and RzMTV2 clustered with Rosellinia necatrix megatotivirus 1 and Picoa juniperi megatotivirus 1 in a clade; and RzYnV1 and RzYnV2 clustered with four other yadonushiviruses in a separated subclade.

### 3.7. Genome Organization and Phylogenetic Analysis of Putative Members of the Proposed Family Tulasviridae

**Figure 7:**
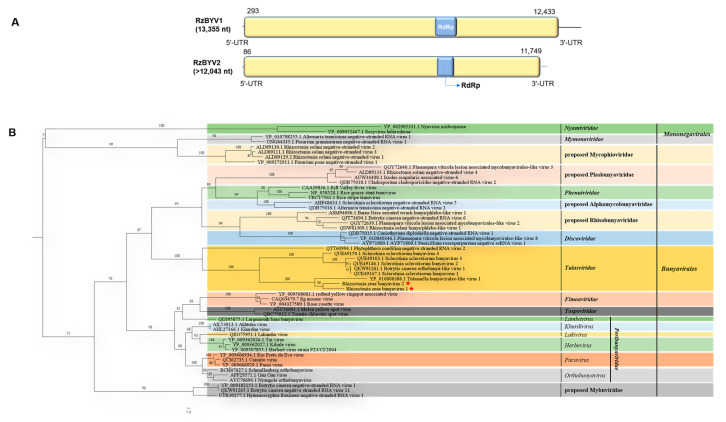
Genome organization of Rhizoctonia zeae bunyavirus 1 (RzBYV1) and Rhizoctonia zeae bunyavirus 2 (RzBYV2) and maximum likelihood tree depicting the relationships of the predicted amino acid (aa) sequences of RNA-dependent RNA polymerase (RdRp) of assembled sequences with similarities to members of the two orders (*Bunyavirales* and *Mononegavirales*). (**A**) Genome organizations of RzBYV1 and RzBYV2. Open reading frames (ORFs) are shown as boxes. One conserved domain in Bunya_RdRp (RdRp) is shown in blue boxes. (**B**) Maximum likelihood phylogenetic tree was constructed based on the aa sequences of the core conserved domain of RdRp of RzBYV1, RzBYV2, and 52 other representative viruses of the two orders (*Bunyavirales* and *Mononegavirales*) using IQ-TREE with the best-fit model “VT+F+R5”. Numbers on the branches indicate the percent bootstrap support from 1000 replicates. The red stars indicate the position of RzBYV1 and RzBYV2.

Contig900 and contig3951, related to two novel mycoviruses, were obtained from metatranscriptome data, which were tentatively designated as Rhizoctonia zeae bunyavirus 1 (RzBYV1) and Rhizoctonia zeae bunyavirus 2 (RzBYV2), respectively. The complete genome sequence of RzBYV1, whose full length was 13,355 nt with a 34.7% G+C content (GenBank accession no. OQ559671), was obtained using the RACE method. The 5′- and 3′-UTRs of RzBYV1 were 292 nt and 922 nt in length, respectively. One ORF of 4046 aa (molecular mass: 453.726 kDa) is predicted from the genome sequence of RzBYV1 and contains a conserved domain of Bunya_RdRp (pfam04196, 6.28 × 10^−5^) (Figure 7A). The near full length of RzBYV2 is 12,043 nt (GenBank accession no. OQ559681). One single ORF of 3887 aa (molecular mass: 469.866 kDa) is predicted from the sequence of RzBYV2 and also contains the conserved domain of RzBYV1 of Bunya_RdRp (pfam04196, 1.10 × 10^−6^). The genome organization of RzBYV1 and RzBYV2 is similar to that of Sclerotinia sclerotiorum bunyavirus 1, Sclerotinia sclerotiorum bunyavirus 2, Sclerotinia sclerotiorum bunyavirus 3, Sclerotinia sclerotiorum bunyavirus 4, Tulasnella bunyavirales-like virus 1, Botrytis cinerea orthobunya-like virus 1, and Phytophthora condilina negative-stranded RNA virus 2, all of which belong to the family *Tulasviridae* [11].

A BLASTp search showed that the complete aa sequence of ORF of RzBYV1 shared the closest identity (31.86%) with that of RdRp of Agrocybe praecox tulasvirus 1, and the complete aa sequence of ORF of RzBYV2 shared the highest identity (31.65%) with that of RdRp of Tulasnella bunyavirales-like 1. Multiple alignments of the aa sequences of RdRp of RzBYV1, RzBYV2, and ten other related members showed that seven conserved motifs (A–F and H) with an SDD tripeptide within motif C were found (Figure S7), which is the same as the characteristics of members within the family *Tulasviridae*. A phylogenetic tree was constructed based on the core region of the putative RdRp protein of RzBYV1, RzBYV2, and 52 other mycoviruses within the two orders, *Bunyavirales* and *Mononegavirales*, indicating that RzBYV1 and RzBYV2 clustered with the members of the family *Tulasviridae* in a separate clade (Figure 7B).

### 3.8. Confirmation of Mycoviruses Contributing to the Assembly of Virus Particles in D40 and Observations of Virus Particles

RT-PCR was conducted to confirm the viruses from the extracted virion suspension contributing to the assembly of virus particles. As a result, RzYnV1, RzYnV2, RzMTV1, RzMTV2, RzRV1, RzRV2, and RzYkV2 present in strain D40 were identified to be responsible for assembling virions, while sequence evidence for the nine other mycoviruses (RzYkV1, RzHV1, RzHV2, RzOLV1, RzOLV2, RzGFV1, RzBYV2, RzBYV1, and RzMBV1) was not obtained from extracted virion suspension (Figure S8).

Further confirmation of viral infection was obtained through observation of virions. Spherical or pleomorphic particles around 80 nm in diameter and pleomorphic particles around 50 nm in diameter were observed (Figure S9A,B). For RzYnV1 and RzYnV2, a type of spherical particle of approximately 40 nm in diameter was observed, as expected for yadonushiviruses (Figure S9B,C) [33].

## 4. Discussion

In this study, sixteen novel mycoviruses co-infecting *R. zeae* strain D40 were identified and molecularly characterized. Moreover, full genome sequences of six mycoviruses (RzHV1, RzOLV1, RzOLV2, RzRV1, RzRV2, and RzBYV1) were obtained. Among these sixteen mycoviruses, seven mycoviruses (RzHV1 and RzHV2 within the family *Hypoviri-dae*, RzYkV1 and RzYkV2 within the family *Yadokariviridae*, RzOLV1 and RzOLV2 within the family *Botourmiaviridae*, and RzGFV1 within the family *Gammaflexiviridae*) contained +ssRNA genome, seven mycoviruses (RzRV1, RzRV2, RzMBV1, RzMTV1, RzMTV2, RzYnV1, and RzYnV2) within the order *Ghabrivirales* contained dsRNA genome, and two mycoviruses (RzBYV1 and RzBYV2) within the family *Tulasviridae* contained −ssRNA genome. According to the classification criteria designated by ICTV, RzHV1, RzHV2, RzYkV1, RzYkV2, RzOLV1, and RzOLV2 were identified to be novel species. Due to species demarcation criteria, they were not applicable on the website of ICTV. Based on genome organization, aa sequence identity, and phylogenetic analysis, the ten other mycoviruses were also speculated to be novel species. To the best of our knowledge, it is the first detailed study of mycoviruses infecting *R. zeae*.

Metatranscriptome sequencing combined with RT-PCR has revealed the genome structures of many mycoviruses and has helped to provide a better understanding of the co-infection of mycoviruses [8,26,34]. As the virosphere in fungi is tremendous and needs more exploration, new species of mycoviruses can possibly be discovered using metatranscriptome sequencing. However, the limitation of metatranscriptome sequencing methods utilized in this study is that only viral sequences containing the conserved domains previously known to be associated with virus genomes can be retrieved, which may overlook mycovirus sequences related to the putative multipartite fragments and sequences lacking conserved region compared with discovered mycoviruses [35,36].

Co-infection of mycoviruses in a single strain is common in diverse fungi [7]. There are many reports documenting a single strain co-infected by +ssRNA viruses only or by both +ssRNA viruses and dsRNA viruses simultaneously [2,23,37,38,39]. The mixed infection of mycoviruses containing +ssRNA, dsRNA, and −ssRNA genome types in a single strain is rarely reported [12]. In the present study, it is the first report of mycoviruses containing three genome types (+ssRNA, dsRNA, and −ssRNA) that co-infect a single strain of *Rhizoctonia*. Co-infection patterns of viruses in fungi are various, indicating the complexity of mycovirosphere in a single strain. Furthermore, co-infection of mycoviruses in fungi will provide a platform for discoveries of virus–virus interactions, including synergistic, antagonistic, and mutualistic interactions between unrelated viruses [40].

Reported members of the family *Hypoviridae* possess one or two ORFs [41]. However, in our study, two hypoviruses (RzHV1 and RzHV2) were identified to possess four discontinuous ORFs. With the exception of RdRp, no other conserved domains were found in RzHV1, while three conserved domains, including one RdRp domain and two Hel domains (DEAD (Hel1) and SF2_C_RHA (Hel2)), were identified in RzHV2. The phenomenon of two helicases co-existing in a single virus has been reported in fusariviruses, deltaflexiviruses, and some plant viruses [12,23]. Recent studies reported that two helicase domains located in the same mycovirus might indicate the horizontal gene transfer event or gene duplication [11,12]. However, more studies are needed to confirm whether a recombination genome or gene duplication event happened in RzHV2 or not. Interestingly, conserved catalytic motif GDD was conserved in both RzHV1 and RzHV2. However, in most hypoviruses, GDD was substituted with SDD [42,43]. Based on the results of phylogenetic analysis, it is worth noting that RzHV1 and RzHV2 do not belong to the established genera in the family *Hypoviridae*. Moreover, the genome organization of RzHV1 and RzHV2 is significantly different from previously reported hypoviruses. Therefore, we propose to establish one new genus in the family *Hypoviridae* to accommodate RzHV1, RzHV2, and other related members. Further studies on the exploitation of novel hypoviruses are needed to describe the molecular properties of viruses in the family *Hypoviridae* in more detail.

*Yadokariviridae* is a newly approved family containing +ssRNA genome by ICTV, which received approval on December 2022 (https://ictv.global/report/chapter/yadokariviridae/yadokariviridae, accessed on28 March 2023) [44]. The first yadokarivirus, namely YKV1, was discovered from the fungus Rosellinia necatrix, co-infecting with YnV1 [33]. Since then, several yadokariviruses were identified from *Sclerotinia sclerotiorum*, *Picoa juniperi*, *Plasmopara veticola*, *Penicillium aurantiogriseum*, and *Aspergillus homomorphus* [45,46,47,48,49]. In addition to the RdRp domain located in the central region of the single polyprotein of yadokariviruses, a 2A-like peptide motif (DxExNPG ↓ P, where ‘x’ = any aa and ‘↓’ indicates the cleavage site) is present in the C-proximal region, which is considered to be involved in ‘ribosome skip’ [31,50]. Using multiple alignments of aa sequences, a 2A-like motif was identified in RzYkV1, RzYkV2, and other yadokariviruses. The phylogenetic analysis indicated that RzYkV1 and RzYkV2 clustered with betayadokariviruses. The species demarcation criteria of new yadokariviruses from ICTV involve both phylogenetic position and nucleotide or aa sequence identity. Taken together, based on the analysis of genome organization, low homology identity (BLASTp identities of 45.16% and 47.04% for RzYkV1 and RzYkV2, respectively), and phylogenetic analysis, RzYkV1 and RzYkV2 are recognized to be new members of the genus Betayadokarivirus within the family *Yadokariviridae*.

The family *Botourmiaviridae* accommodates 12 genera, namely *Ourmiavirus*, *Botoulivirus*, *Magoulivirus*, *Scleroulivirus*, *Penoulivirus*, *Betabotoulivirus*, *Betarhizolivirus*, *Betascleroulivirus*, *Deltascleroulivirus*, *Epsilonscleroulivirus*, *Gammascleroulivirus*, and *Rhizoulivirus* [51]. We identified two novel ourmia-like mycoviruses (RzOLV1 and RzOLV2) in strain D40, both of which contained a single ORF with a conserved RdRp domain. The genome of RzOLV1 is specific in the termini of the G tract and its complementary structure, which may inhibit the digestive activity of exonucleases from helping viruses escape from antiviral defenses, and similar terminal structures have been found in the genome of some mitoviruses, narnaviruses, and ourmiaviruses [32,52,53,54]. RzOLV1 clustered with the members of the genus *Magoulivirus* within the family *Botourmiaviridae*. RzOLV2 clustered with members of the genus *Rhizoulivirus*, which was generated from the host *Rhizoctonia* and harbored most botoumiaviruses identified from *Rhizoctonia*, in accord with the presumption of co-evolution with host [14]. Complete aa sequence identities of putative RdRp proteins between different viruses in the genus Rhizoulivirus are less than 90%. Based on the highest identities (BLASTp identities of 43.65% and 44.95%, respectively, for RzOLV1 and RzOLV2) of the complete aa sequences of RdRp and phylogenetic analysis, RzOLV1 and RzOLV2 are proposed to be new members of the family *Botourmiaviridae*.

The family *Gammaflexiviridae*, as well as four other families, namely *Tymoviridae*, *Alphaflexiviridae*, *Betaflexiviridae*, and *Gammaflexiviridae*, belongs to the order *Tymovirales* (the ninth report of the ICTV: https://ictv.global/report_9th, accessed on 28 March 2023). The host range of members in the order *Tymovirales* includes plants and fungi, but gammaflexiviruses have only been found to infect filamentous fungi [36]. Previously discovered members of the family *Gammaflexiviridae* contain one single segment with two or three ORFs encoding RdRp, CP, HP, or MP. Except for Pistacia-associated flexivirus 1, the genomes of other gammaflexiviruses, including Entoleuca gammaflexivirus 1, Entoleuca gammaflexivirus 2, and Botrytis virus F, contain discontinuous ORFs. The genome of RzGFV1 consists of a single segment with three ORFs. Among them, ORF1 and ORF2 overlapped, and there was a discontinuous pattern between ORF2 and ORF3. Additionally, a viral methyltransferase-conserved domain was identified in ORF1, and the helicase domain and RdRp domain were identified in ORF2, while ORF3 was relatively short with unknown function. Previous reports indicated that the presence of methyltransferase domain might suggest the capping of the 5'-end of the genomic RNA [55]. The species demarcation criteria from ICTV to identify new members of the family *Gammaflexiviridae* are not applicable. However, based on genome organization, complete aa sequence identity of RdRp (BLASTp highest identity of 34.77%), and phylogenetic analysis, RzGFV1 was proposed to be a new member of the family *Gammaflexiviridae*.

*Ghabrivirales* is an order hosting seven families containing the dsRNA genome, including *Megabirnaviridae*, Yadonushiviridae, Megatotiviridae, *Chrysoviridae*, *Quadriviridae*, *Totiviridae*, and Fusagraviridae [49]. In the present study, members of the three families, *Megabirnaviridae*, Yadonushiviridae, and Megatotiviridae, belonging to the order *Ghabrivirales* and the proposed family Phlegiviridae closely related to the order *Ghabrivirales* were identified from strain D40. The genomes of RzRV1, RzRV2, RzMBV1, RzMTV1, RzMTV2, RzYnV1, and RzYnV2 identified from the order *Ghabrivirales* in this study consist of one single segment containing two discontinuous ORFs, encoding CP and RdRp, respectively.

Phlegiviridae was proposed by Picarelli et al. in 2019 [14]. A conserved domain of SMC was found in RzRV2 and was named chromosome segregation ATPase or structural maintenance of chromosomes domain, which is considered to play central roles in regulating order chromosome dynamics from bacteria to humans [56,57]. In addition, this unique domain was also found in Corynespora cassiicola fusarivirus 1, Fusarium graminearum hypovirus 2, and Rhizoctonia solani dsRNA virus 11 (phlegivirus) [57,58,59]. The phylogenetic analysis of members of the order *Ghabrivirales* indicated that phlegiviruses were closely related to megabirnaviruses in a separate clade. Previous reports have revealed the phylogenetic correspondence of phlegivirus, megabirnavirus, and totivirus [14,57,60]. Based on similar genomic characteristics, phylogenetic analysis, and multiple alignments of seven mycoviruses and members of the four families (Phlegiviridae, *Megabirnaviridae*, Megatotiviridae, and Yadonushiviridae), the members of the proposed family Phlegiviridae were closely related to the members in the order *Ghabrivirales*, inferring that this family might be a member of the order *Ghabrivirales*. Phlegiviridae is a newly proposed family without sufficient properties to be referred to. Two novel phlegiviruses with full-length genomes were identified in this study, which will further help classify the proposed family Phlegiviridae.

*Megabirnaviridae* is a member of the order *Ghabrivirales*. Most megabirnaviruses have two dsRNA segments, and the larger segment contains two ORFs with a ribosomal frameshifting mechanism (a shifty heptamer) [61]. However, the genome of Rhizoctonia solani RNA virus HN008 (a member closely related to megabirnaviruses) and RzMBV1 do not contain shifty heptamer but contain two discontinuous ORFs [57,62]. However, the species demarcation criteria of the family *Megabirnaviridae* from ICTV have not been defined. Taken together, based on the unique organization of RzMBV1, low BLASTp identity of complete aa sequence of RdRp (the highest of 37.97%), and phylogenetic analysis, RzMBV1 is proposed to be a new member of the family *Megabirnaviridae*.

Megatotiviridae is a member of the order *Ghabrivirales* as well and was proposed by Arjona-Lopez et al. in 2018 [10]. To date, two mycoviruses, Picoa juniperi megatotivirus 1 (PjMTV1) and Rosellinia necatrix megatotivirus 1 (RnMTV1), have been reported to be members of the proposed family Megatotiviridae. In our study, there are two members (RzMTV1 and RzMTV2) belonging to the proposed family Megatotiviridae. The genome length of RzMTV2 with complete ORFs is 12,984 nt, which is similar to the genome of PjMTV1 (13,313 nt in length) and RnMTV1 (12,430 nt in length). However, the genome length of RzMTV1 with complete ORFs is 5909 nt, being significantly different from these three megatotiviruses. The identification of RzMTV1 and RzMTV2 enriched the members of the family Megatotiviridae. Taken together, based on genome organization, low homology identity (the highest BLASTp identities of 45.05% and 41.68% for complete aa sequences of RdRp of RzMTV1 and RzMTV2, respectively), and phylogenetic analysis, RzMTV1 and RzMTV2 are speculated to be novel members of the proposed family Megatotiviridae.

Yadonushiviridae is another member of the order *Ghabrivirales*. We failed, however, to determine the complete ORF encoding CP of RzYnV1 and RzYnV2, which may be due to the low titer of the two mycoviruses. However, the results of the confirmation of these two mycovirus-assembling virions, which can be observed under TEM, further confirmed the presence of RzYnV1 and RzYnV2. Additionally, the size of spherical particles of RzYnV1 and RzYnV2 were around 40 nm in diameter, which were expected to be similar to the size of virions of yadonushiviruses [33].

Up to now, reports on particle morphologies of RzRV1, RzRV2, RzMTV1, and RzMTV2 are still absent. In this study, the particles of 40 nm, 50 nm, and 80 nm in diameter were possibly the morphology of these four viruses. However, more evidence is needed to support this presumption. The species demarcation criteria of the four families (Phlegiviridae, *Megabirnaviridae*, Yadonushiviridae, and Megatotiviridae) were not defined. Based on the results of genome organization and phylogenetic analysis, RzRV1, RzRV2, RzMTV1, RzMTV2, RzYnV1, RzYnV2, and RzMBV1 are proposed to be new members of these four families.

*Bunyavirales* is a large virus order consisting of fourteen families recognized by ICTV (https://ictv.global/taxonomy, accessed on 28 March 2023). However, recent studies expanded the taxonomy of members within the order *Bunyavirales*, including Mycophenuiviridae, Rhisobunyaviridae, etc. [34]. In our study, two viruses (RzBYV1 and RzBYV2) related to bunyaviruses shared the highest complete aa sequence identities with that of RdRp of Agrocybe praecox tulasvirus 1 (31.86%) and Tulasnella bunyavirales-like 1 (31.65%), respectively. Seven motifs found in RzBYV1 and RzBYV2 are in correspondence with the conserved motifs found in the members within the two families (Mycophenuiviridae and *Tulasviridae*) [11]. Collectively, based on genome organization, multiple alignment analysis, and phylogenetic analysis, RzBYV1 and RzBYV2 are proposed to be new members of the family *Tulasviridae*.

In this study, we described the molecular properties of sixteen novel RNA mycoviruses co-infecting a single strain of *R. zeae* strain D40 and obtained full-length genomes of six novel mycoviruses. To the best of our knowledge, no gammaflexiviruses, megatotiviruses, or tulasviruses have previously been reported from *Rhizoctonia*, and thus, this study expands the diversity of mycoviruses present in *Rhizoctonia*. Moreover, proposals of newly discovered viruses are raised in this study, including the proposal of one new genus in the family *Hypoviridae*, which further enriches the diversity of mycovirus taxonomy. In conclusion, our findings shed light on the variations of mycovirus diversity in *Rhizoctonia* and provide new insights into viral taxonomy and evolution and the co-infection of different mycoviruses. However, the virus–virus interactions in D40 and virus–host interactions are still unclear and need further experimental verification in the future.

## Figures and Tables

**Table 1:** The information of the sixteen mycoviruses (Rhizoctonia zeae hypovirus 1, Rhizoctonia zeae hypovirus 2, Rhizoctonia zeae yadokarivirus 1, Rhizoctonia zeae yadokarivirus 2, Rhizoctonia zeae ourmia-like virus 1, Rhizoctonia zeae ourmia-like virus 2, Rhizoctonia zeae gammaflexivirus 1, Rhizoctonia zeae dsRNA virus 1, Rhizoctonia zeae dsRNA virus 2, Rhizoctonia zeae megabirnavirus 1, Rhizoctonia zeae megatotivirus 1, Rhizoctonia zeae megatotivirus 2, Rhizoctonia zeae yadonushivirus 1, Rhizoctonia zeae yadonushivirus 2, Rhizoctonia zeae bunyavirus 1, and Rhizoctonia zeae bunyavirus 2) present in *Rhizoctonia zeae* strain D40.

Virus name	Accession Number	Length (nt)	BLASTx Best Match	Identity of aa (%)	E-Value	Genome Type	Family/Order
Rhizoctonia zeae hypovirus 1	OQ559666	12,558	Mycosphaerella hypovirus A	31.50	3 × 10^−170^	+ssRNA	*Hypoviridae*
Rhizoctonia zeae hypovirus 2	OQ559672	13,543	Ceratobasidium hypovirus A	46.14	0	+ssRNA	*Hypoviridae*
Rhizoctonia zeae yadokarivirus 1	OQ559673	6755	Aspergillus homomorphus yadokarivirus 1	45.16	2 × 10^−154^	+ssRNA	*Yadokariviridae*
Rhizoctonia zeae yadokarivirus 2	OQ559674	7116	Aspergillus homomorphus yadokarivirus 1	47.04	2 × 10^−177^	+ssRNA	*Yadokariviridae*
Rhizoctonia zeae ourmia-like virus 1	OQ559667	2959	Leucocoprinus ourmiavirus B	44.95	2 × 10^−86^	+ssRNA	*Botourmiaviridae*
Rhizoctonia zeae ourmia-like virus 2	OQ559668	4221	Agaricus bisporus virus 15	43.17	1 × 10^−140^	+ssRNA	*Botourmiaviridae*
Rhizoctonia zeae gammaflexivirus 1	OQ559675	6971	Botrytis virus F	34.44	3 × 10^−129^	+ssRNA	*Gammaflexiviridae*
Rhizoctonia zeae RNA virus 1	OQ559669	12,529	Rhizoctonia solani dsRNA virus 18	42.52	0	dsRNA	proposed Phlegiviridae
Rhizoctonia zeae RNA virus 2	OQ559670	10,448	Rhizoctonia solani dsRNA virus 18	42.43	0	dsRNA	proposed Phlegiviridae
Rhizoctonia zeae megabirnavirus 1	OQ559676	7933	Rhizoctonia solani megabirnavirus 2	37.44	1 × 10^−131^	dsRNA	*Megabirnaviridae*
Rhizoctonia zeae megatotivirus 1	OQ559677	5909	Rosellinia necatrix megatotivirus 1	44.19	0	dsRNA	proposed Megatotiviridae
Rhizoctonia zeae megatotivirus 2	OQ559678	12,923	Rosellinia necatrix megatotivirus 1	41.08	0	dsRNA	proposed Megatotiviridae
Rhizoctonia zeae yadonushi virus 1	OQ559679	4421	Ceratobasidium virus A	48.28	0	dsRNA	proposed Yadonushiviridae
Rhizoctonia zeae yadonushi virus 2	OQ559680	4463	Ceratobasidium virus A	48.48	0	dsRNA	proposed Yadonushiviridae
Rhizoctonia zeae bunyavirus 1	OQ559671	13,355	Agrocybe praecox tulasvirus 1	32.08	7 × 10^−119^	−ssRNA	*Tulasviridae*
Rhizoctonia zeae bunyavirus 2	OQ559681	12,043	Tulasnella bunyavirales-like 1	31.41	8 × 10^−101^	−ssRNA	*Tulasviridae*

## Data Availability

The sequences reported in the present manuscript have been deposited in the GenBank database under accession numbers OQ559666, OQ559667, OQ559668, OQ559669, OQ559670, OQ559671, OQ559672, OQ559673, OQ559674, OQ559675, OQ559676, OQ559677, OQ559678, OQ559679, OQ559680, and OQ559681. Raw data of the metatranscriptome sequencing have been deposited in the Sequence Read Archive (SRA) database under the accession number SAMN33386006.

## Supplementary Materials

The following supporting information can be downloaded at https://www.mdpi.com/article/10.3390/jof10010030/s1. Figure S1: The double-stranded RNA (dsRNA) extracted from mycelia of *Rhizoctonia zeae* strain D40 was stained with ethidium bromide and performed in 1.5% agarose gel electrophoresis. M: 250 bp DNA Ladder; D40: dsRNA extracted from *R. zeae* strain D40. Figure S2: Multiple alignments of amino acid (aa) sequences of RNA-dependent RNA polymerase (RdRp) of Rhizoctonia zeae hypovirus 1 (RzHV1) and Rhizoctonia zeae hypovirus 2 (RzHV2) and helicase (Hel) of RzHV2. (**A**) Multiple aa alignments of RdRp of RzHV1, RzHV2, and five representative hypoviruses, in which eight conserved motifs (I–VIII) are indicated. (**B**) Multiple aa alignments of Hel of RzHV2 and six other representative hypoviruses, in which six conserved motifs (I-VI) are indicated. “*” indicates identical amino acids, “:” indicates highly chemically similar amino acids, and “.” indicates less chemically similar amino acids. Sequence information for all viruses selected is listed in Table S4. Figure S3: Amino acid (aa) sequence properties of RzYkV1 and RzYkV2. (**A**) Multiple alignments of aa sequences of RNA-dependent RNA polymerase (RdRp) encoded by RzYkV1, RzYkV2, and eleven other representative yadokariviruses, in which seven motifs (A–G) are indicated. (**B**) Multiple alignments of aa sequences of 2A-like motifs in the members of the family *Yadokariviridae*. “*” indicates identical amino acids, “:” indicates highly chemically similar amino acids, and “.” indicates less chemically similar amino acids. Sequence information for all viruses selected is listed in Table S4. Figure S4: Multiple alignments of amino acid (aa) sequences of RNA-dependent RNA polymerase (RdRp) encoded by RzOLV1, RzOLV2, and seven other representative botourmiaviruses, in which eight motifs (I–VIII) are indicated. “*” indicates identical amino acids, “:” indicates highly chemically similar amino acids, and “.” indicates less chemically similar amino acids. Sequence information for all viruses selected is listed in Table S4. Figure S5: Multiple alignments of amino acid (aa) sequences of RNA-dependent RNA polymerase (RdRp) encoded by RzGFV1 and five other representative gammaflexiviruses, in which eight motifs (I–VIII) are indicated. “*” indicates identical amino acids, “:” indicates highly chemically similar amino acids, and “.” indicates less chemically similar amino acids. Sequence information for all viruses selected is listed in Table S4. Figure S6: Multiple alignments of amino acid (aa) sequences of RNA-dependent RNA polymerase (RdRp) encoded by RzRV1, RzRV2, RzMTV1, RzMTV2, RzYnV1, RzYnV2, RzMBV1, and eight other representative ghabriviruses, in which eight motifs (I–VIII) are indicated. “*” indicates identical amino acids, “:” indicates highly chemically similar amino acids, and “.” indicates less chemically similar amino acids. Sequence information for all viruses selected is listed in Table S4. Figure S7: Multiple alignments of amino acid (aa) sequences of RNA-dependent RNA polymerase (RdRp) encoded by RzBYV1, RzBYV2, and ten other representative bunyaviruses, in which seven motifs (A–F, H) are indicated. “*” indicates identical amino acids, “:” indicates highly chemically similar amino acids, and “.” indicates less chemically similar amino acids. Sequence information for all selected viruses is listed in Table S4. Figure S8: Products amplified by reverse transcription polymerase chain reaction (RT-PCR) using primers designed based on nucleotide sequences of each mycovirus and visualized using 1% agarose gel electrophoresis analysis to confirm the mycoviruses contributing to the assembly of virions. Figure S9: Observation of virions with transmission electron microscope (TEM). (**A**) Blue arrows point to the virus particles with about 50 nm in diameter, and purple arrows point to the virus particles with about 80 nm in diameter. (**B**) The purple arrow points to the virus particles with about 80 nm in diameter, and the red arrow points to the virus particles with about 40 nm in diameter. (**C**) The red arrow points to the virus particles that are about 40 nm in diameter. Table S1: Primer pairs used to identify the sixteen mycoviruses (Rhizoctonia zeae hypovirus 1, Rhizoctonia zeae hypovirus 2, Rhizoctonia zeae yadokarivirus 1, Rhizoctonia zeae yadokarivirus 2, Rhizoctonia zeae ourmia-like virus 1, Rhizoctonia zeae ourmia-like virus 2, Rhizoctonia zeae gammaflexivirus 1, Rhizoctonia zeae dsRNA virus 1, Rhizoctonia zeae dsRNA virus 2, Rhizoctonia zeae megabirnavirus 1, Rhizoctonia zeae megatotivirus 1, Rhizoctonia zeae megatotivirus 2, Rhizoctonia zeae yadonushivirus 1, Rhizoctonia zeae yadonushivirus 2, Rhizoctonia zeae bunyavirus 1, and Rhizoctonia zeae bunyavirus 2) present in *Rhizoctonia zeae* strain D40 and to confirm the mycoviruses that assemble into virions by reverse transcript polymerase chain reaction (RT-PCR). Table S2: Primer pairs designed based on the results from metatranscriptome sequencing to validate less assembled regions with obscure N bases and/or fragments/gaps of the sixteen mycoviruses (Rhizoctonia zeae hypovirus 1, Rhizoctonia zeae hypovirus 2, Rhizoctonia zeae yadokarivirus 1, Rhizoctonia zeae yadokarivirus 2, Rhizoctonia zeae ourmia-like virus 1, Rhizoctonia zeae ourmia-like virus 2, Rhizoctonia zeae gammaflexivirus 1, Rhizoctonia zeae dsRNA virus 1, Rhizoctonia zeae dsRNA virus 2, Rhizoctonia zeae megabirnavirus 1, Rhizoctonia zeae megatotivirus 1, Rhizoctonia zeae megatotivirus 2, Rhizoctonia zeae yadonushivirus 1, Rhizoctonia zeae yadonushivirus 2, Rhizoctonia zeae bunyavirus 1, and Rhizoctonia zeae bunyavirus 2) present in *Rhizoctonia zeae* strain D40 by reverse transcript polymerase chain reaction (RT-PCR). Table S3: Primers used to determine the untranslated regions (UTR) of the six mycoviruses (Rhizoctonia zeae hypovirus 1, Rhizoctonia zeae ourmia-like virus 1, Rhizoctonia zeae ourmia-like virus 2, Rhizoctonia zeae dsRNA virus 1, Rhizoctonia zeae dsRNA virus 2, and Rhizoctonia zeae bunyavirus 1) present in *Rhizoctonia zeae* strain D40. Table S4: The information on reference viruses retrieved from GenBank database (National Center for the Biotechnology Information) and used to conduct multiple alignments.
