# Supplementary material for: Sixteen Novel Mycoviruses Containing Positive Single-Stranded RNA, Double-Stranded RNA, and Negative Single-Stranded RNA Genomes Co-Infect a Single Strain of Rhizoctonia zeae
Source: J Fungi (Basel). 2023 Dec 31;10(1):30. doi: 10.3390/jof10010030 (PMC10817634; doi:10.3390/jof10010030)
Supplement: Supplementary file 1 [file jof-10-00030-s001.zip › Table S1.pdf]

Supplementary Table S1. Primer pairs used to identify the sixteen mycoviruses (Rhizoctonia zeae hypovirus 1, Rhizoctonia zeae hypovirus 2, Rhizoctonia zeae yadokarivirus 1, Rhizoctonia zeae yadokarivirus 2, Rhizoctonia zeae ourmia-like virus 1, Rhizoctonia zeae ourmia-like virus 2, Rhizoctonia zeae gammaflexivirus 1, Rhizoctonia zeae dsRNA virus 1, Rhizoctonia zeae dsRNA virus 2, Rhizoctonia zeae megabirnavirus 1, Rhizoctonia zeae megatotivirus 1, Rhizoctonia zeae megatotivirus 2, Rhizoctonia zeae yadonushivirus 1, Rhizoctonia zeae yadonushivirus 2, Rhizoctonia zeae bunyavirus 1, and Rhizoctonia zeae bunyavirus 2) present in *Rhizoctonia zeae* strain D40 and to confirm the mycoviruses that assemble into virions by reverse transcript-polymerase chain reaction (RT-PCR).

Confirmed viruses	Primer name	Sequence (5'-3')	Contig ID	Fragment size (nt)	Annealing temperature (°C)
Rhizoctonia zeae hypovirus 1	201-13F	TCTGCTCGCCATTGGTTACTACTTCC	contig201	1016	62
	201-13R	CCAGTTGCCATCATAGACTTCATCGT			60
Rhizoctonia zeae hypovirus 2	1743-3F	CCCGAGTTTGGCTTCCATTTGT	contig1743	988	60
	1743-3R	GGACCGCAGACTGTTGTTGACC			60
Rhizoctonia zeae yadokarivirus 1	764-2F	TCACTAATCCTACCCTACCTCATAACGC	contig764	744	61
	764-2R	TCTGATTCAAGCGAAGTGCCAAG			58
Rhizoctonia zeae yadokarivirus 2	351-5F	ATCGGATTCACGACTCAGCG	contig351	826	57
	351-5R	GGCGGGAGAAGAAGGACAG			59
Rhizoctonia zeae ourmia-like virus 1	69-1F	CCTGCCCATTTCAGTTTAA	contig69	619	53
	69-1R	GCTGTGAGGCTCGGATT			55
Rhizoctonia zeae ourmia-like virus 2	132-1F	TGTGGGATGAGCAGTATGAT	contig132	821	53
	132-1R	CCTCGCAGCAAGGTTAGT			55
Rhizoctonia zeae gammaflexivirus 1	797-7F	ACTACAAGCACTCGGCCAGCAA	contig797	978	60
	797-7R	TCAGGAAGGCGTCGAACAGGA			60
Rhizoctonia zeae RNA virus 1	75YZ-F	ATGAACGGTGACATAGCGAAGAAACA	contig75	963	61
	75YZ-R	TCCTCCTCCGAATGAACCAGCA			61
Rhizoctonia zeae RNA virus 2	620YZ-F	TGGACCGAATCTAACAGGTTGAAGCA	contig620	484	62
	620YZ-R	GACCACAACTGGCACCGAACGAC			62
Rhizoctonia zeae megabirnavirus 1	79486-F	GCCGAACTCCGAACCCGAACCT		840	63

	79884-R	CGATCTCATCCCTCACTCGCTCCCT	contig7030 + contig9453 + contig4186		66
Rhizoctonia zeae megatotivirus 1	6273-7F	GGCTTGATGACTTTGCTG	contig6273	1173	53
	6273-7R	GGGACAGGGTCTTCTTACTC			57
Rhizoctonia zeae megatotivirus 2	496-F	AGTTCGGTGGTGCTGAGGCG	contig496	873	62
	496-R	CGTTGGCGTGATTGGAGGTGTT			60
Rhizoctonia zeae yadonushi virus 1	3587-1F	CGAACCCGTCCCGCTCATA	contig3587	492	60
	3587-1R	AGGGCGTCGGCTCGAACAA			60
Rhizoctonia zeae yadonushi virus 2	3588-5F	TTTTCTGAGTTCCTACGCTACG	contig3588	725	56
	3588-5R	ACACGGGCATCATTCTGG			55
Rhizoctonia zeae bunyavirus 1	900YZ-1F	TCTGCGGCTCGTAAGGAA	contig900	721	57
	900YZ-1R	TGTCAGGAGTGAAAGATGGGAT			56
Rhizoctonia zeae bunyavirus 2	3951YZ-3F	CGTTCGGTTGTTGATAGAGC	contig3951	610	56
	3951YZ-3R	TTGATAAAGGAGGCGTTGG			54
