# Supplementary material for: Sixteen Novel Mycoviruses Containing Positive Single-Stranded RNA, Double-Stranded RNA, and Negative Single-Stranded RNA Genomes Co-Infect a Single Strain of Rhizoctonia zeae
Source: J Fungi (Basel). 2023 Dec 31;10(1):30. doi: 10.3390/jof10010030 (PMC10817634; doi:10.3390/jof10010030)
Supplement: Supplementary file 1 [file jof-10-00030-s001.zip › Table S2.pdf]

Supplementary Table S2. Primer pairs designed based on the results from metatranscriptome sequencing to validate less assembled region with obscure N bases and fragments/gaps of the sixteen mycoviruses (Rhizoctonia zeae hypovirus 1, Rhizoctonia zeae hypovirus 2, Rhizoctonia zeae yadokarivirus 1, Rhizoctonia zeae yadokarivirus 2, Rhizoctonia zeae ourmia-like virus 1, Rhizoctonia zeae ourmia-like virus 2, Rhizoctonia zeae gammaflexivirus 1, Rhizoctonia zeae dsRNA virus 1, Rhizoctonia zeae dsRNA virus 2, Rhizoctonia zeae megabirnavirus 1, Rhizoctonia zeae megatotivirus 1, Rhizoctonia zeae megatotivirus 2, Rhizoctonia zeae yadonushivirus 1, Rhizoctonia zeae yadonushivirus 2, Rhizoctonia zeae bunyavirus 1, and Rhizoctonia zeae bunyavirus 2) present in *Rhizoctonia zeae* strain D40 by reverse transcript-polymerase chain reaction (RT-PCR).

Virus name	Primer name	Sequence (5'-3')	Contig ID	Region of RT-PCR product at viral genome (nt)	Annealing temperature (℃)
Rhizoctonia zeae hypovirus 1	201-1F	TAAGTAACCCTGACGAGCCA	contig201	118-640	55
	201-1R	GATGCGAAACGCCAACAC			55
	201-2F	TAGCAGGGTATGGCAACG		402-1179	55
	201-2R	AAAGGCGAAGAGTGGGAG			55
	201-3F	CTCTTCGCCTTTGGACTA		1168-1849	53
	201-3R	TACCAGGACGGCTGTGAT			55
	201-4F	AGAGCGAGCCGAAGATG		1266-4923	55
	201-4R	CAATGGCGAGCAGAGGT			55
	201-5F	GTCTATCACAAGGCGGGAGT		3674-6654	57
	201-5R	GCTGGCAGATGGGAAAA			52
	201-9F	GGTGGAAACCCTGACAGCGTGAT		1811-2695	61
	201-9R	CCTAGCATCCTCCCAGCATTCG			61
	201-10F	GGGAAGTGGCTGATGCGTTGTA		2640-3533	60
	201-10R	TTGATTGATTTGCGTCGAAGGTG			56
	201-11F	ATGGAGTTCACCTTCGACGCAAAT		3503-4443	58
	201-11R	TCAAGCCTCGTCAACACCCACA			60
	201-12F	GTTTGCCGCCCGAGTTCGT		4258-5086	60

	201-12R	TTTAGCCGCAGCATCAATCCC			58
	201-13F	TCTGCTCGCCATTGGTTACTACTTCC		4910-5926	62
	201-13R	CCAGTTGCCATCATAGACTTCATCGT			60
	201-14F	TTGAGGTCCTTTGGGTGTTGCG		5841-6835	60
	201-14R	CCTTCGTGGGAGGGAGGCTATG			63
	201-16F	CCGTTTCGGGTCCCTTAGTGC		7351-8423	61
	201-16R	TGATCTTCCGGTATTCAGTTCTTGTCC			60
	201-17F	GCGATCCGACCTTGGAGCAG		8214-9210	62
	201-17R	TTCCCGATTGGTAAGAAAGATGTTGT			56
	201-18F	GGGGCAACGGCACATTCATT		9071-10,105	57
	201-18R	TTTGGACTTGGAGCCTCATTTCAG			58
	201-19F	ACTGAAATGAGGCTCCAAGTCCAAA		10,081-11,083	58
	201-19R	ACTTCCTCCGTCTTCAAGCAATAACTC			60
	201-20F	AGGAGTTATTGCTTGAAGACGGAGGA		11,057-11,967	60
	201-20R	GAGGCGGTGTAGTCTGTCGTGAAA			61
	201-21F	ATCACTATCTGAGCCAGTAACACCCG		11,734-12,440	61
	201-21R	ACGCGAATTGCGACAAACCC			57
	1743-1F	TAAAGACTTGGACGGCGGATGG		226-1304	60
	1743-1R	GGTTTGCGTGAAGAAGGGAGGA			60
	1743-2F	TTTGGAGGCAGTCTGAGATGGA		1140-1901	58
	1743-2R	GCCCTTGGTGTAATGTAGCGAAT			58
Rhizoctonia zeae hypovirus 2	1743-3F	CCCGAGTTTGGCTTCCATTTGT	contig1743	1708-2695	58
	1743-3R	GGACCGCAGACTGTTGTTGACC			61
	1743-5F	CAGCACGGAAGAAGAACCTA		3153-4068	55
	1743-5R	TCTCCACCAGGACAGTATAAATC			56
	1743-6F	CTAGTGCAGAACACCCACCTC		3954-4896	60

	1743-6R	GCTCCAATGCCAACCTTTT			53
	1743-10F	CCGACGCAACTACACCACA		6578-7580	57
	1743-10R	GTTTAGCATTTATCGCCACATT			52
	1743-13F	AGGCGCACTACAACCAGC		8841-9647	57
	1743-13R	CGTAATCGTCTTCCGACTCAA			56
	1743-14F	GGGGATAACCCAACCAGG		9497-10,380	57
	1743-14R	TGAGACTCCAGCCATTTCG			55
	1743-16F	TCACATCTTTCTTTGGCATTG		11,127-12,167	52
	1734-16R	TTGTTGGTGTCGCTTGGA			53
	1743-18F	ATCCTCCTCGGTAATCCTTTC		12,757-13,356	56
	1743-18R	TGACGCATTATTGAGTCTTGG			54
	764-1F	TTGCGTCAGCAGTGTTCT		269-1217	53
	764-1R	AGGCGTTATGAGGTAGGG			55
	764-2F	TCACTAATCCTACCCTACCTCATAACGC		1188-1932	61
	764-2R	TCTGATTCAAGCGAAGTGCCAAG			58
	764-4F	CCACTTTGACTCCCGATACCCTT		2811-3717	60
	764-4R	CATTTGGTCGTGGCAGTGAGGT			60
Rhizoctonia zeae yadokarivirus 1	764-5F	CCGCAATACCGTCTTAGGAGGA	contig764	3604-4482	60
	764-5R	GGATCGTATCTGTCTGTAGAAAGTGGC			61
	764-7F	ACAGGAATGTTAGCCGACAAGCG		5096-5956	60
	764-7R	CCAATGAAGGCGGTCAGCAC			60
	764-8F	TTCTCGCAAACGTCGGTTGAA		5784-6561	56
	764-8R	TCGGACCTAAATAAGGTGGTCACTAAA			58
	351-1F	GCCCTGATTTGAGGTTCG		166-926	55
Rhizoctonia zeae yadokarivirus 2	351-1R	GAGGAGCATCGGGACATT	contig351		55
	351-2F	ACGACGGACCCATCACCA		722-1799	57

	351-2R	GAAGCAAACCGACGGAAGT			55
	351-3F	GACTTCCGTCGGTTTGCTT		1780-2755	55
	351-3R	GGTGGCGGTCCTTGAGATA			57
	351-4F	CACGAGCCTGCCTTCCTT		2524-3561	57
	351-4R	TTCGGTTGCGACGGTTTT			53
	351-5F	ATCGGATTCACGACTCAGCG		3416-4242	57
	351-5R	GGCGGGAGAAGAAGGACAG			59
	351-6F	CGCCTCCTCCTACCACTTC		4061-4950	55
	351-6R	CTTGTTGCGGATACCTTCG			55
	351-7F	CCAACGGCTGAAGGTGAT		4762-5701	55
	351-7R	GAAGGGAGGTGCTGGAGAC			60
	351-8F	TGGAAACCACCGTCATCTATT		5615-6531	54
	351-8R	CAGCCTTTACACTGCGAACTA			56
	351-9F	GAGGATACGCCGACTGCA		6299-7006	57
	351-9R	CGAAGGGATTGAGATAAGGAAG			56
Rhizoctonia zeae ourmia-like virus 1	69-1F	CCTGCCCATTTCAGTTTAA	contig69	35-653	51
	69-1R	GCTGTGAGGCTCGGATT			55
	69-2F	AAGTCCAGGTAGCCATTCA		440-1983	53
	69-2R	AGGGTTTCCACCGTTTT			50
	69-3F	TGTGCTGGAGAAATAGGGA		1930-2887	53
	69-3R	AGGAAGTCTGGTTTGAGGG			55
Rhizoctonia zeae ourmia-like virus 2	132-1F	TGTGGGATGAGCAGTATGAT	contig132	133-954	53
	132-1R	CCTCGCAGCAAGGTTAGT			55
	132-4F	TGGAATCCTTTTGTCGTC		3877-4200	50
	132-4R	CATTTATTATGAACCCCGTAG			52

	132-5F	CGAAAGATGCGTAGTGTCATACTCGTACTGT		744-1683	62
	132-5R	CAAGATAAACGAAGACTCGGCTCCAGAT			61
	132-6F	ATCTGGAGCCGAGTCTTCGTTTATCTTG		1656-2527	61
	132-6R	GCTTGACGCTTGCCACCTGTTGA			61
	132-7F	GCTCAACAGGTGGCAAGCGTCAA		2503-3543	61
	132-7R	GGGAGGTCAATATGGGCTGGGAT			61
	132-8F	CCTGCGGAATCCTGGCTCG		3384-3981	62
	132-8R	GAACATCTTCGCTGGGGAAATCAT			58
	797-2F	CCCCATCTGTCCAATCAAACCC		128-433	60
	797-2R	TGCAGGGACCATAGATCCGAGAA			60
	797-3F	CTTTCCCGCCTCCCTGACCT		994-1578	53
	797-3R	GTCGTTCTCGTCCGTTGCCTC			55
	797-4F	CGAGGCAACGGACGAGAACG		1557-2327	60
	797-4R	TGGCTGAACGAGCGGAGGAG			60
	797-5F	GTCGTGGTCCAGCACCTTCG		2050-3117	62
	797-5R	AGCGGGTGTCGGCTTGGTA			62
Rhizoctonia zeae gammaflexivirus 1	797-6F	CACTTCGATGCGGAGCCAGAG	contig797	2943-3981	62
	797-6R	GGCGGTTGATGACCAGGAGG			60
	797-7F	ACTACAAGCACTCGGCCAGCAA		3720-4698	60
	797-7R	TCAGGAAGGCGTCGAACAGGA			60
	797-8F	CTTCGCAAAGCGGATCAAGC		4601-5629	57
	797-8R	TCGGCACCACCGTCAAACAA			57
	797-9F	TACGAGGAGTTGCTTCCGTTGTTT		5592-6113	58
	797-9R	AGAAGAGGTGGAGAACCGTGGG			61
	797-10F	CTCTTTCTCCTTCCTCCTCACCTTTC		404-1169	61

	797-10R	CGACCTGCGTCATTCTTCGTGT			60
Rhizoctonia zeae RNA virus 1	75YZ-F	ATGAACGGTGACATAGCGAAGAAACA	contig75	7488-8451	61
	75YZ-R	TCCTCCTCCGAATGAACCAGCA			61
Rhizoctonia zeae RNA virus 2	620YZ-F	TGGACCGAATCTAACAGGTTGAAGCA	contig620	6900-7394	62
	620YZ-R	GACCACAACTGGCACCGAACGAC			62
	620YZ-2F	GGTCCACGATGCCCGAAAGG		4342-5218	60
	620YZ-2R	GAACCCGCCGAAGAACCACAGA			61
Rhizoctonia zeae megabirnavirus 1	7030-4F	GTTCCACGAGCGGTACTCCTCATCACTG	contig7030 + contig9453 + contig4186	1480-2208	65
	7030-4R	CAGCCCGACGTACACCTCCTCAACC			66
	7030-5F	CGCTGTTCGCTCGGGCTTCTGGTA		2114-3223	65
	7030-5R	GATGGGCCGCATGATGCTGTTGG			63
	RzMBV1-gap3-1F	CCGCAGGCATGGCGACATCA		3262-3921	63
	RzMBV1-gap3-1R	TCCGTCAGCTCAGTTATCCGCAGGTT			66
	9486-2F	CGTCATCACCCAGGTCTCACTCGTC		4378-5154	65
	9486-2R	CGCTCGGGTCATAAACCACTCTGC			63
	9486-3F	ATCCCTCACTCGCTCCCTTCCACT		5010-5844	63
	9486-3R	CCGAACTCCGAACCCGAACCTG			63
	RzMBV1-gap2F	TTCCGAGCACCGCTTCACCG		5655-6020	62
	RzMBV1-gap2R	ACTGATTCAACAGCCCAATGCGAGA			60
	9486-4F	CAGGTTCGGGTTCGGAGTTCGG		5822-6568	63
	9486-4R	GCGGCAGCATTCCACAATCAAGG			61
	9486-5F	TGGCGATCAATCAGTGGAGTGGC		6563-7578	61

	9486-5R	AAGCGCATCAGTTAGGCGTGGC			61
Rhizoctonia zeae megatotivirus 1	6273-1F	AGGCTCGCATCATTTCA	contig6273	110-774	50
	6273-1R	AGGTCAGTCCTGGTGGC			57
	6273-6F	CCACCTGGCTTACCTTAG		3462-4624	55
	6273-6R	TATCAATCCTGGCACCC			52
Rhizoctonia zeae megatotivirus 2	496-2F	GGCACATTCACTTCAATAATCAACTTCG	contig496	8260-8802	61
	496-2R	CAGCGGTTCGTACCTGTACTCTTTCA			63
Rhizoctonia zeae yadonushi virus 1	3587-1F	CGAACCCGTCCCGCTCATA	contig3587	53-545	60
	3587-1R	AGGGCGTCGGCTCGAACAA			60
	3587-2F	TGACACGGCTAACGAACT		411-915	53
	3587-2R	GAAAGGGAAGGAAGAAGG			53
	3587-3F	CACCTCCACTCCCATCCA		15-413	57
	3587-3R	AGTTCGTTAGCCGTGTCATT			53
	3587-4F	CTTCCCTTCTTCCTTCCCTT		819-1792	55
	3587-4R	CAATCGTGTAGCAGTAGTGGC			57
	3587-5F	TGGTCCGCCACTACTGCT		1766-2720	57
	3587-5R	TCTTGCTGCGTGTTGTTGA			53
	3587-6F	CCAGCGACATTGACGACC		2564-3453	57
	3587-6R	GAGCAGGAAGGCAGAAGTG			57
	3587-7F	AGTTCACTTCTGCCTTCCTG		3431-4101	55
	3587-7R	ATACCGCCTCAAAGACACC			55
	3587-8F	CCCAAATTGCCCTAGAAA		3836-4404	50
	3587-8R	GAGCGTGAAAGTGAGATGAG			55
Rhizoctonia zeae yadonushi virus 2	3588-3F	CGCCACATCATCAAGAACA	contig3588	1226-2172	53
	3588-3R	CCGTAAGCGACAGCAGAG			57
	3588-5F	TTTTCTGAGTTCCTACGCTACG		2957-3681	56

	3588-5R	ACACGGGCATCATTCTGG			55
	3588-6F	CAAGGAGAAGACCGTTGAGA		3457-4436	55
	3588-6R	GAGCGTGAAAGTGAGGAATG			55
Rhizoctonia zeae bunyavirus 1	900YZ-1F	TCTGCGGCTCGTAAGGAA	contig900	4560-5280	57
	900YZ-1R	TGTCAGGAGTGAAAGATGGGAT			56
Rhizoctonia zeae bunyavirus 2	3951YZ-1F	AGACTGTTACATCCCTGAGCC	contig3951	2104-2824	57
	3951YZ-1R	TTTCCGCATCTGAGGTTTC			54
