# Supplementary material for: Sixteen Novel Mycoviruses Containing Positive Single-Stranded RNA, Double-Stranded RNA, and Negative Single-Stranded RNA Genomes Co-Infect a Single Strain of Rhizoctonia zeae
Source: J Fungi (Basel). 2023 Dec 31;10(1):30. doi: 10.3390/jof10010030 (PMC10817634; doi:10.3390/jof10010030)
Supplement: Supplementary file 1 [file jof-10-00030-s001.zip › Table S3.pdf]

Supplementary Table S3. Primers used to determine the untranslated regions (UTR) of the six mycoviruses (Rhizoctonia zeae hypovirus 1, Rhizoctonia zeae ourmia-like virus 1, Rhizoctonia zeae ourmia-like virus 2, Rhizoctonia zeae dsRNA virus 1, Rhizoctonia zeae dsRNA virus 2, and Rhizoctonia zeae bunyavirus 1) present in *Rhizoctonia zeae* strain D40.

Amplification target	Primer name	Sequence (5'-3')	Annealing temperature (℃)
5'-UTR of Rhizoctonia zeae hypovirus 1 (RzHV1)	RzHV1-5'-1	GCCCTGTCGGAAGTCAGCGTAG	63
	RzHV1-5'-2	GGATGATGTTGGAGCCCTGTCG	61
	RzHV1-5'-3	CCCTGTCGGAAGTCAGCGTAGTG	63
3'-UTR of RzHV1	RzHV1-3'-1	TGAGCAGGGAAGGAGTCTAGGGC	63
	RzHV1-3'-2	CGCATCCCGAGGCATTCAA	57
	RzHV1-3'-3	AACGCATCCCGAGGCATTCA	57
5'-UTR of Rhizoctonia zeae ourmia-like virus 1 (RzOLV1)	RzOLV1-5'-5	GGTGAAGCCCACGAGCGGTAAA	61
	RzOLV1-5'-1	CGAGCGGTAAAGCGGAAGGTG	61
3'-UTR of RzOLV1	RzOLV1-3'-2	TGGGAGACCGACACGAGGAGAAG	63
	RzOLV1-3'-1	CGACACGAGGAGAAGGAATTGGG	61
5'-UTR of Rhizoctonia zeae ourmia-like virus 2 (RzOLV2)	RzOLV2-5'-1	GCGTCGCAATTCCACCCTGTG	61
3'-UTR of RzOLV2	RzOLV2-3'-1	CTCGGAGGTGACGGTGGAAGGA	63
	RzOLV2-3'-2	TCCTGCGGAATCCTGGCTCG	62
	RzOLV2-3'-4	CGAGACGCTTGATGCCGAGGTT	61
5'-UTR of Rhizoctonia zeae RNA virus 1 (RzRV1)	RzRV1-5'-2	GGCACGGCAACAGGGAGGAA	62
3'-UTR of RzRV1	RzRV1-3'-2	TGGGAGACTCATCGCCTGTCCTT	61
5'-UTR of Rhizoctonia zeae RNA virus 2 (RzRV2)	RzRV2-5'-3	GGCTTGCACCCGCAGTTTGG	62
3'-UTR of RzRV2	RzRV2-3'-2	TCAGCGGGATCAACGGTGGC	62
	RzRV2-3'-6	AGGAGGCTGTTGCTGCCGATGT	61
5'-UTR of Rhizoctonia zeae bunyavirus 1 (RzBYV1)	RzBYV1-5'-1	ATGGGCCGTACCTCACCAC	59
	RzBYV1-5'-2	AATGGGCCGTACCTCACC	55
3'-UTR of RzBYV1	RzBYV1-3'-1	CGCCAGGCCGACCTGTAGCTCTAT	65
