# Supplementary material for: Sixteen Novel Mycoviruses Containing Positive Single-Stranded RNA, Double-Stranded RNA, and Negative Single-Stranded RNA Genomes Co-Infect a Single Strain of Rhizoctonia zeae
Source: J Fungi (Basel). 2023 Dec 31;10(1):30. doi: 10.3390/jof10010030 (PMC10817634; doi:10.3390/jof10010030)
Supplement: Supplementary file 1 [file jof-10-00030-s001.zip › Table S4.pdf]

Supplementary Table S4. The information of reference viruses retrieved from GenBank database (National Center for the Biotechnology Information) and used to conduct multiple alignments.

Family/Order	Name of reference virus	GenBank accession number
	Sclerotinia sclerotiorum hypovirus 1 (SsHV1) [a]	AEL99352.1
	Botrytis cinerea hypovirus 1 (BcHV1)	QBA69887.1
Hypoviridae	Cryphonectria hypovirus 4 (CHV4)	YP 138519.1
	Macrophomina phaseolina hypovirus 1 (MpHV1)	ALD89099.1
	Rhizoctonia solani hypovirus 2 (RsHV2)	QDW92700.1
	Aspergillus homomorphus yadokarivirus 1 (AhoYV1)	AZT88626.1
	Aspergillus foetidus slow virus 2 (AfV-S2)	CCD33025.1
	Penicillium aurantiogriseum foetidus-like virus (PaFIV1)	YP 009182156.1
	Plasmopara viticola lesion associated yadokari virus 1 (PvLaYkV1)	QHD64758.1
	Yado-kari virus 1 (RnYkV1)	YP 009551451.1
Yadokariviridae	Yado-kari virus 3 (RnYkV3)	BBB86810.2
	Yado-kari virus 2 (RnYkV2)	AVD68673.2
	Yado-kari virus 4 (RnYkV4)	BBB86805.1
	Sclerotinia sclerotiorum yadokari virus 1 (SsYkV1)	UCR17164.1
	Fusarium poae mycovirus 2 (FpMyV2)	YP 009272910.1
	Picoa juniperi yado-kari virus 1 (PjYV1)	QOI17269.1
	Rhizoctonia solani ourmia-like virus 4 (RsOLV4)	QDW65429.1
	Magnaporthe oryzae ourmia-like virus (MOLV1)	YP 009667033.1
	Lentinula edodes magoulivirus virus 1 (LeOLV1)	QOX06059.1
Botourmiaviridae	Magnaporthe oryzae botourmiavirus 9 (MoBV9)	UIX55986.1
	Ourmia melon virus (OuMV)	ACF16360.1
	Botrytis ourmia-like virus (BOLV)	YP 009182165.1
	Sclerotinia sclerotiorum ourmia-like virus 4 (SsOLV4)	QHG11400.1

Gammaflexiviridae	Sclerotinia sclerotiorum gammaflexivirus 1 (SsGFV1)	QUE49151.1
	Botrytis virus F (BVF)	NP 068549.1
	Entoleuca gammaflexivirus 1 (EnFV1)	AVD68667.2
	Pistacia-associated flexivirus 1 (PAFV1)	QDO72745.1
	Entoleuca gammaflexivirus 2 (EnFV2)	AVD68668.2
proposed Phlegiviridae	Rhizoctonia solani dsRNA virus 18 (RsdsRNA18)	QXI69650.1
	Rhizoctonia fumigata mycovirus (RfMV)	AJE29745.1
proposed Megatotiviridae	Rosellinia necatrix megatotivirus 1 (RnMTV1)	BDB32683.1
	Picoa juniperi megatotivirus 1 (PjMTV1)	QOI17264.1
proposed Yadonushiviridae	Ceratobasidium virus A (CbVA)	AOX47544.1
	Yado-nushi virus 1-A (YnV1)	YP 009551446.1
Megabirnaviridae	Rosellinia necatrix megabirnavirus 1/W779 (RnMBV1)	YP 003288763.1
	Fusarium pseudograminearum megabirnavirus 1 (FpgMBV1)	AYJ09269.1
Bunyavirales	Sclerotinia sclerotiorum bunyavirus 1 (SsBYV1)	QUE49147.1
	Sclerotinia sclerotiorum bunyavirus 2 (SsBYV2)	QUE49146.1
	Sclerotinia sclerotiorum bunyavirus 3 (SsBYV3)	QUE49103.1
	Sclerotinia sclerotiorum bunyavirus 4 (SsBYV4)	QUE49150.1
	Rhizoctonia solani negative-stranded virus 4 (RsNSRV4)	ALD89133.1
	Botrytis cinerea negative-stranded RNA virus 1 (BcNSRV-1)	YP 009182153.1
	Alternaria tenuissima negative-stranded RNA virus 2 (AtNSRV2)	QDB75016.1
	Sclerotinia sclerotiorum negative-stranded RNA virus 5 (SsNSRV5)	AHF48633.1
	Rift Valley fever virus (RVFV)	CAA39836.1
	Fusarium poae negative-stranded virus 2 (FpNSV2)	YP 009272912.1

[a]: Letters followed by Arabic numerals in the parentheses represent the abbreviation of the virus.
