# Supplementary figures and images for: Sixteen Novel Mycoviruses Containing Positive Single-Stranded RNA, Double-Stranded RNA, and Negative Single-Stranded RNA Genomes Co-Infect a Single Strain of Rhizoctonia zeae
Source: J Fungi (Basel). 2023 Dec 31;10(1):30. doi: 10.3390/jof10010030 (PMC10817634; doi:10.3390/jof10010030)

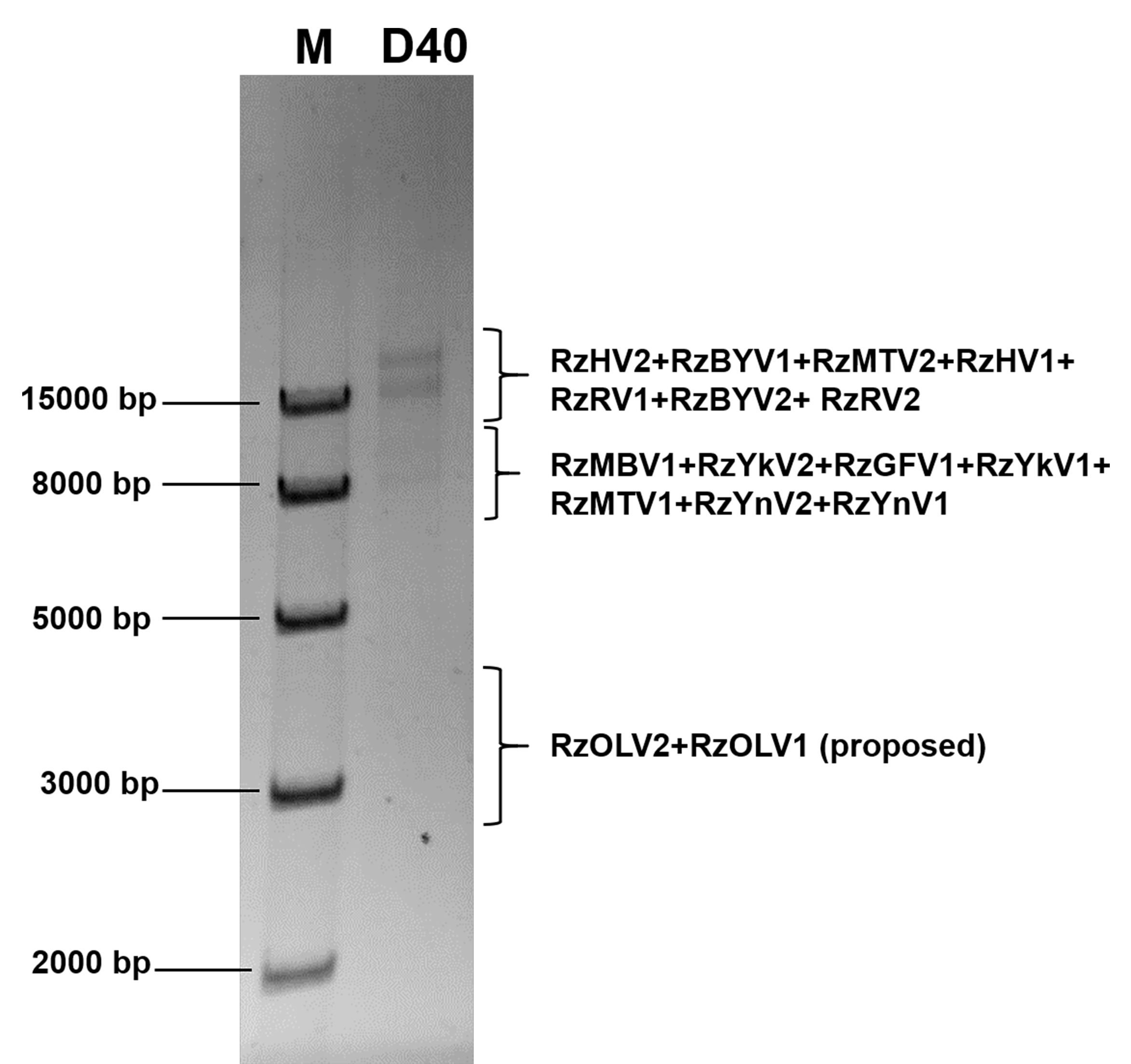

Supplement: Supplementary file 1 [file jof-10-00030-s001.zip › Figure S1.tif]

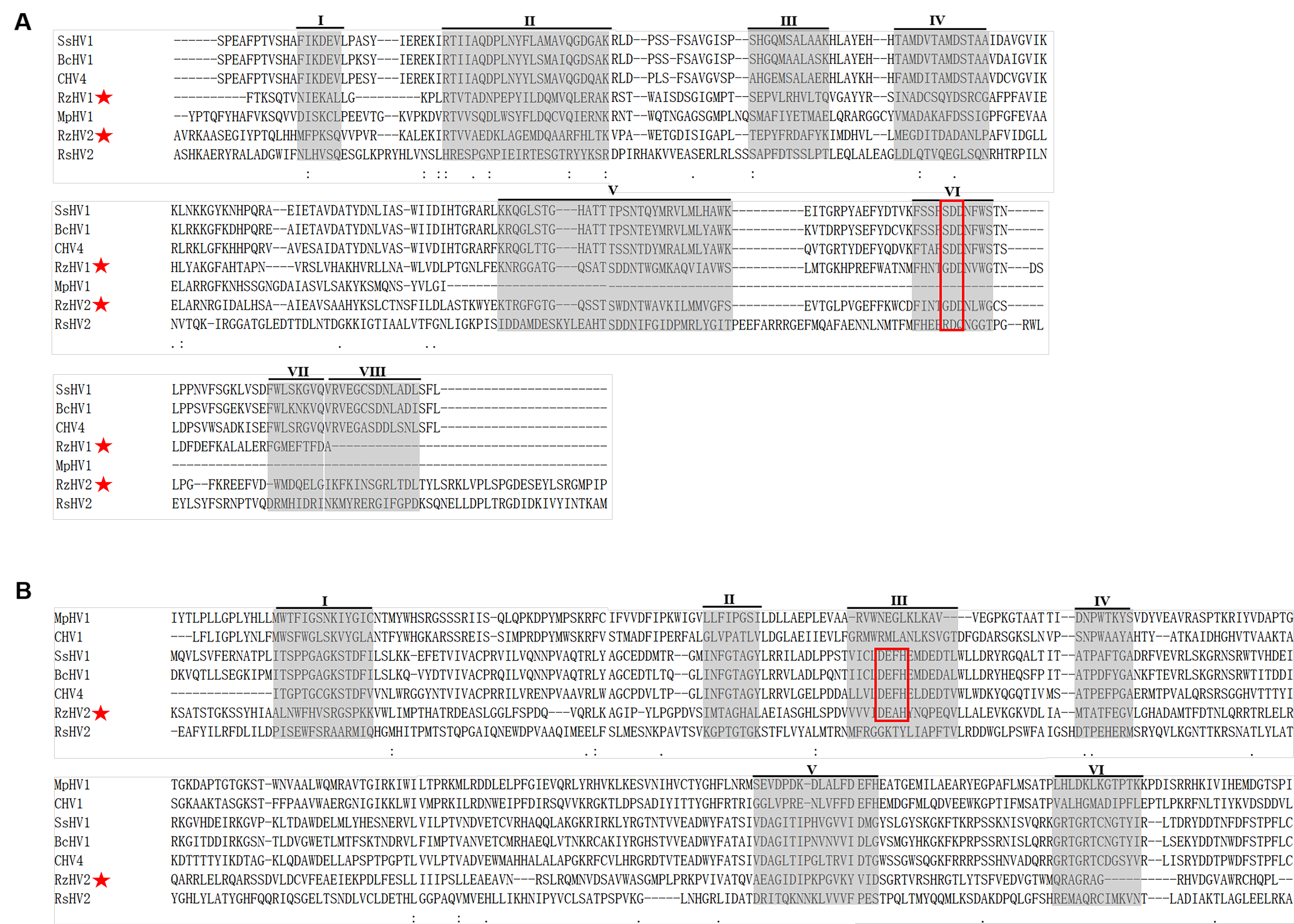

Supplement: Supplementary file 1 [file jof-10-00030-s001.zip › Figure S2.tif]

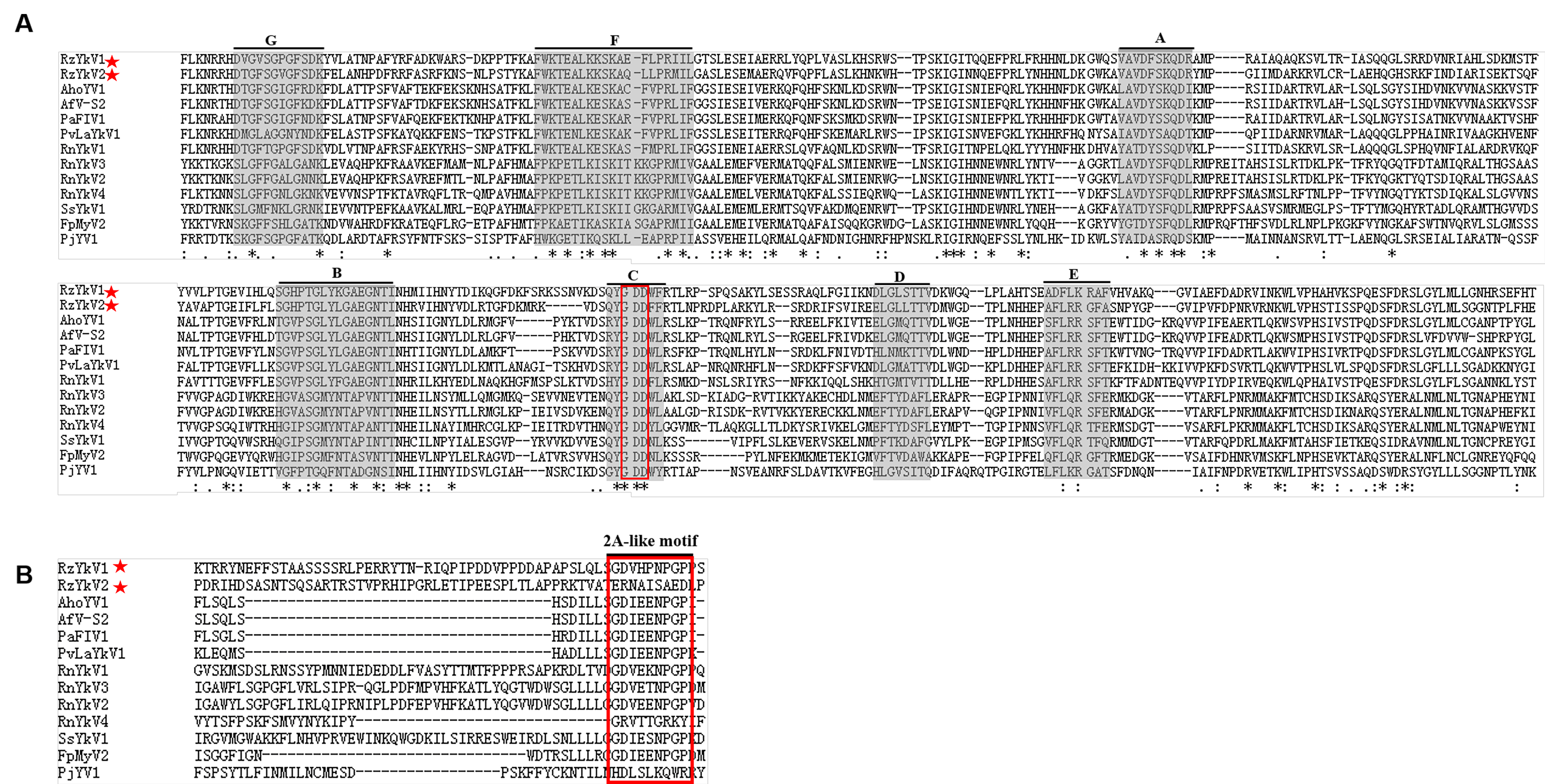

Supplement: Supplementary file 1 [file jof-10-00030-s001.zip › Figure S3.tif]

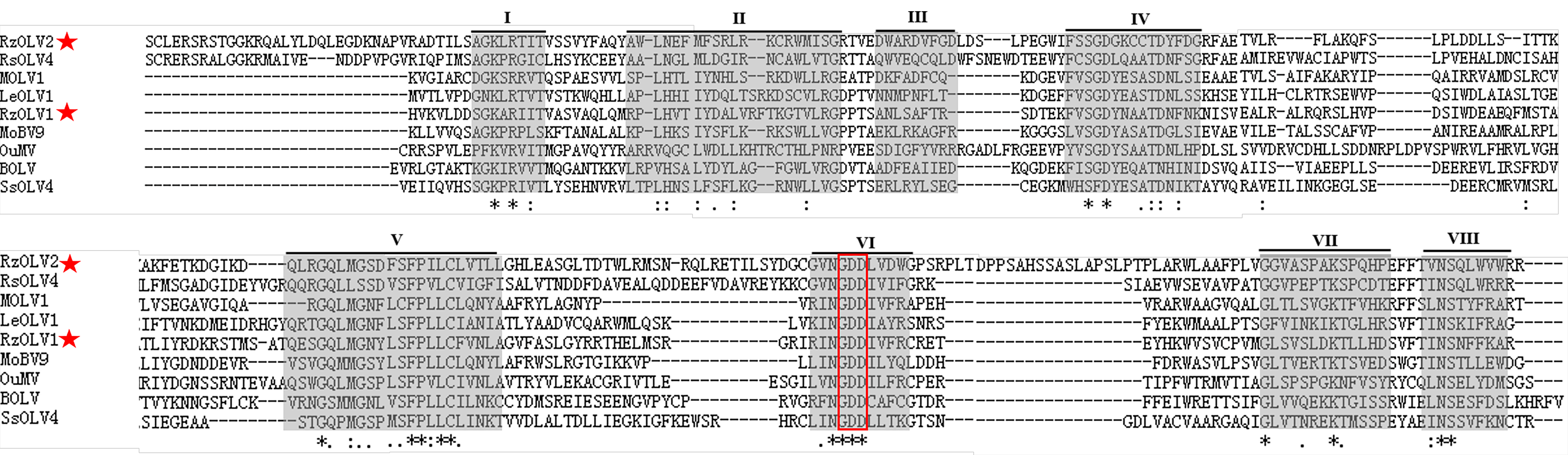

Supplement: Supplementary file 1 [file jof-10-00030-s001.zip › Figure S4.tif]

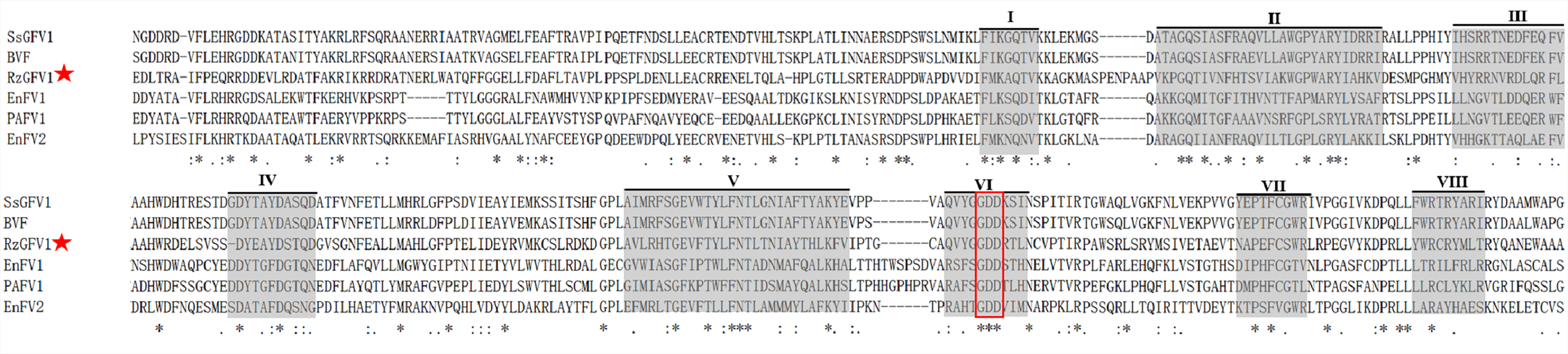

Supplement: Supplementary file 1 [file jof-10-00030-s001.zip › Figure S5.tif]

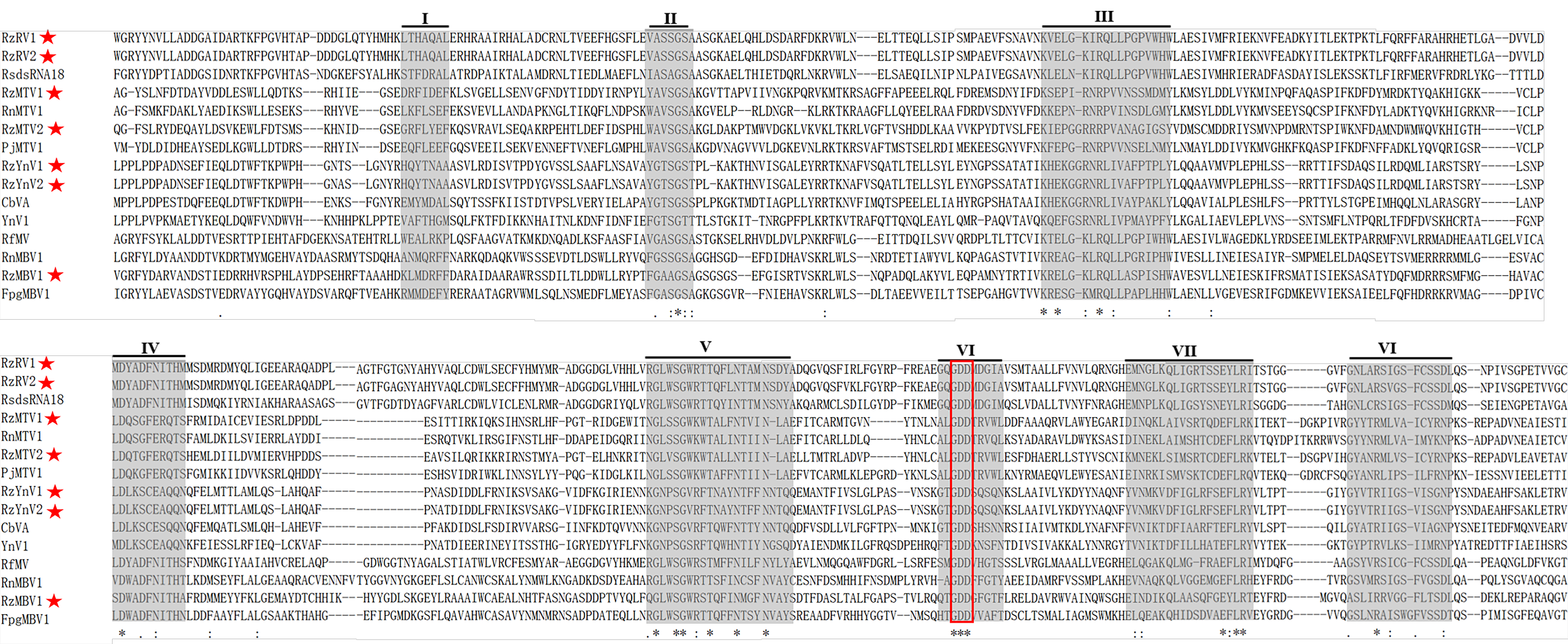

Supplement: Supplementary file 1 [file jof-10-00030-s001.zip › Figure S6.tif]

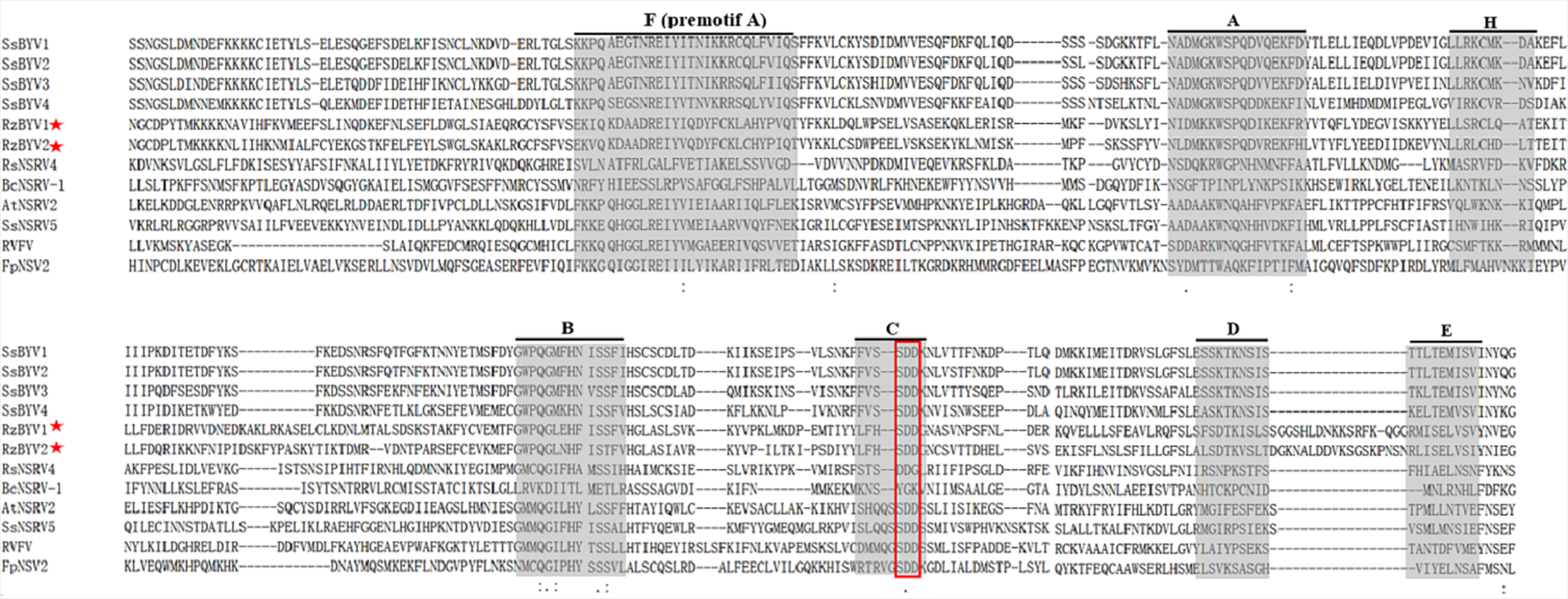

Supplement: Supplementary file 1 [file jof-10-00030-s001.zip › Figure S7.tif]

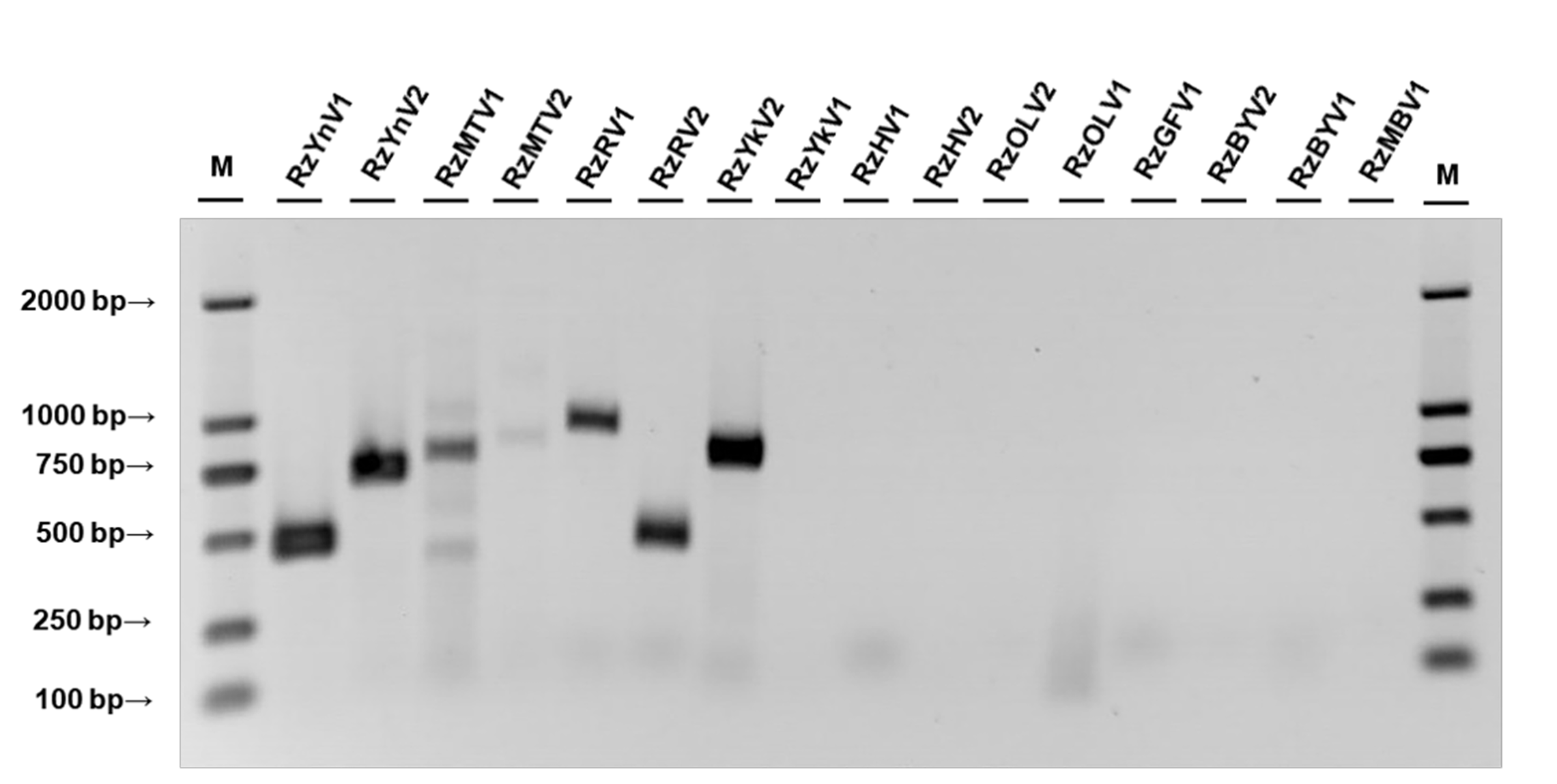

Supplement: Supplementary file 1 [file jof-10-00030-s001.zip › Figure S8.tif]

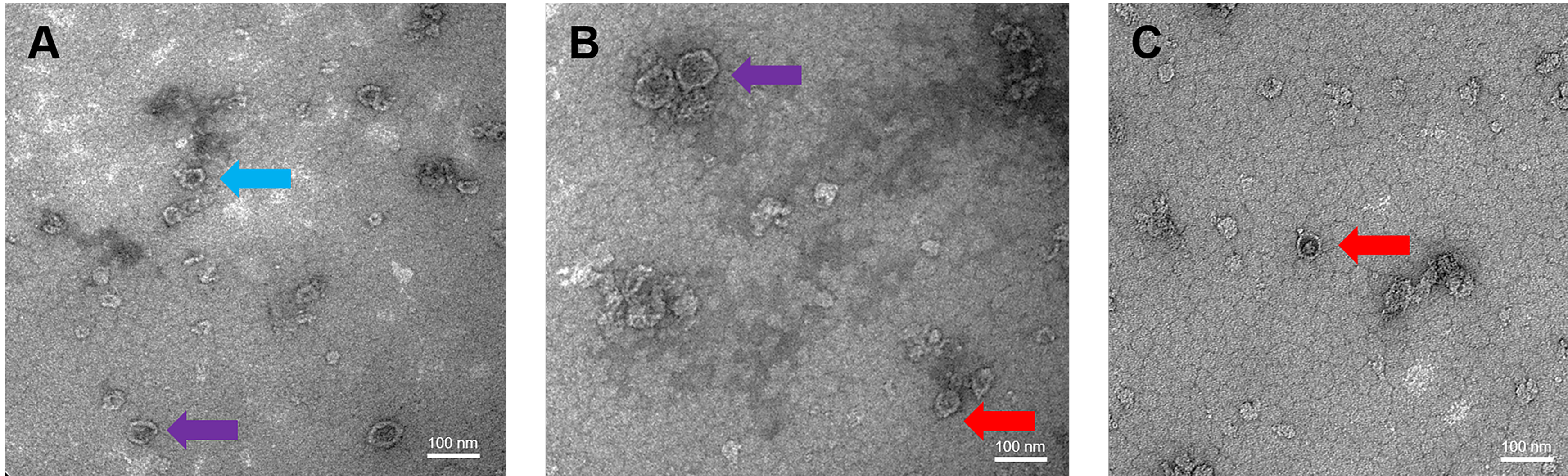

Supplement: Supplementary file 1 [file jof-10-00030-s001.zip › Figure S9.tif]
